# Relation extraction for diet, non-communicable disease and biomarker associations (RECoDe): a CoDiet study

**DOI:** 10.1093/bioinformatics/btag439

**Published:** 2026-08-21

**Authors:** Donghee Choi, Yajie Gu, Kai Qi Zong, Antoine D Lain, Dimitrios Zaikis, Thomas Rowlands, Marek Rei, The CoDiet Consortium, Donghee Choi, Yajie Gu, Kai Qi Zong, Antoine D Lain, Dimitrios Zaikis, Thomas Rowlands, Marek Rei, Tim Beck, Joram M Posma, Tim Beck, Joram M Posma

**Affiliations:** Section of Bioinformatics, Division of Systems Medicine, Department of Metabolism, Digestion and Reproduction, Imperial College London, London, W12 0NN, United Kingdom; School of Computer Science and Engineering, Pusan National University, Busan, 46241, South Korea; Centre for Health Informatics, School of Medicine, University of Nottingham, Nottingham, NG7 2RD, United Kingdom; Section of Bioinformatics, Division of Systems Medicine, Department of Metabolism, Digestion and Reproduction, Imperial College London, London, W12 0NN, United Kingdom; Section of Bioinformatics, Division of Systems Medicine, Department of Metabolism, Digestion and Reproduction, Imperial College London, London, W12 0NN, United Kingdom; School of Informatics, Aristotle University of Thessaloniki, Thessaloniki, 57001, Greece; Centre for Health Informatics, School of Medicine, University of Nottingham, Nottingham, NG7 2RD, United Kingdom; Department of Computing, Imperial College London, London, SW7 2AZ, United Kingdom; CoDiet Project https://www.codiet.eu/; Centre for Health Informatics, School of Medicine, University of Nottingham, Nottingham, NG7 2RD, United Kingdom; Section of Bioinformatics, Division of Systems Medicine, Department of Metabolism, Digestion and Reproduction, Imperial College London, London, W12 0NN, United Kingdom

## Abstract

Diet plays a critical role in human health, with growing evidence linking dietary habits to disease outcomes. However, extracting structured dietary knowledge from biomedical literature remains challenging due to the lack of dedicated relation extraction datasets. To address this gap, we introduce RECoDe, a novel relation extraction (RE) dataset designed specifically for diet, disease, and related biomedical entities. RECoDe captures a diverse set of relation types, including a broad spectrum of positive association patterns and explicit negative examples, with over 5000 human-annotated instances validated by up to five independent annotators. Furthermore, we benchmark various natural language processing (NLP) RE models, including BERT-based architectures and enhanced prompting techniques with locally deployed large language models (LLMs) to improve classification performance on underrepresented relation types. The best performing model was gpt-oss-20B, a locally-deployed open-weight LLM, achieving an F1-score of 61% (macro) for multi-class classification and 89% for binary classification using a hierarchical prompting strategy with a separate reflection step built in. To demonstrate the practical utility of RECoDe, we introduce the Contextual Co-occurrence Summarisation (CoCoS) framework, which aggregates sentence-level relation extractions into document-level summaries and further integrates evidence across multiple documents. CoCoS produces effect estimates consistent with established dietary knowledge, demonstrating its validity as a general framework for systematic evidence synthesis.

*Availability:* The code, models, and dataset are publicly available at https://github.com/omicsNLP/RECoDe.

## 1 Introduction

Diet plays a fundamental role in human health, impacting both the prevention and progression of non-communicable diseases (NCDs). Dietary factors shape disease outcomes through well-defined relationships, including their role in regulating blood pressure (Oude Griep *et al.* 2025), modulating lipid metabolism (Lovegrove 2025), influencing weight management (Pugh *et al.* 2024), and contributing to metabolic changes in ageing (Dagbasi *et al.* 2024).

Systematic reviews play an important role in synthesising dietary evidence. A systematic review is the structured process by which domain experts identify, critically appraise, and synthesise all relevant studies on a given question, so that clinicians, researchers, and policymakers can make informed decisions based on the combined body of evidence rather than on individual studies. This rigorous, transparent, and reproducible approach to evaluating unstructured biomedical literature minimises bias and informs research and policy decisions (Lichtenstein *et al.* 2008). However, the sheer volume of unstructured data makes traditional systematic reviews slow and labour-intensive. Recent advancements in natural language processing (NLP) offer promising, yet still evolving, approaches to automating systematic review processes, enhancing efficiency in screening and extracting relevant evidence (Khraisha *et al.* 2024).

Among these developments, biomedical information extraction (IE)—the task of identifying entities (e.g. foods, diseases) and the relations between them in text—provides the foundation for structuring knowledge from unstructured literature (Yoon *et al.* 2022). Building on such approaches, biomedical knowledge graphs (KGs) provide a scalable framework for integrating diverse insights and uncovering complex associations (Geleta *et al.* 2021). However, diet-disease relations remain under-explored, necessitating dedicated datasets and tailored methodologies to bridge this gap.

Despite advances in biomedical relation extraction (RE), existing datasets predominantly focus on drug-disease and gene-disease interactions, leaving dietary relationships significantly underrepresented. This limitation prevents NLP models from effectively capturing the nuanced effects of diet on health. The lack of structured datasets tailored for diet-disease RE hinders the development of models capable of systematically extracting and analysing these relationships.

Building upon previous efforts in diet-related corpus creation (Lain *et al.* 2025), we expand this work by developing a high-quality, manually annotated dataset, RECoDe, that captures fine-grained relationships between diet, disease, and related bio-entities. Understanding these complex associations is essential for assessing disease risk and outcomes, providing a structured foundation for evaluating dietary risks on disease outcomes (Zheng *et al.* 2022).

To evaluate RECoDe, we benchmark multiple NLP models, including traditional language-model approaches based on BERT (Devlin *et al.* 2019) and recent prompting techniques with locally deployed large language models (LLMs), and compare our method against the recent DiMB-RE framework (Hong *et al.* 2025), which is a benchmark dataset and framework for extracting diet–microbiome relations from biomedical literature. Our analysis highlights the advantages and challenges of leveraging LLMs for diet-disease RE, particularly in handling underrepresented relation types.

Our key contributions include (i) the first dataset for diet-disease-bioentity RE enabling structured dietary knowledge discovery, (ii) a detailed annotation schema that captures complex dietary interactions with high granularity, and (iii) extensive benchmarking using transformer-based models and LLM-guided approaches to improve classification performance on underrepresented relation types. By providing a structured foundation for dietary RE, RECoDe paves the way for more accurate and scalable biomedical NLP applications in nutrition science. We anticipate that our dataset, with over 5000 human-reviewed relations, will serve as a valuable resource for computational health research, facilitating deeper insights into the role of diet in disease mechanisms.

## 2 Data and methods

### 2.1 RECoDe dataset

Existing biomedical RE datasets primarily focus on specific molecular interactions, such as drug-disease (Lim *et al.* 2018), gene-disease (Lee *et al.* 2017), and protein-protein interactions (Lai *et al.* 2023). One of the most comprehensive biomedical RE datasets, BioRED, covers various entity types, including disease, gene, and chemical (Luo *et al.* 2022). However, even BioRED lacks dietary components, limiting its applicability to nutrition research. We use the CoDiet corpus dataset (Lain *et al.* 2025) and from this resource, we selected 109 full-text documents from PubMed Central (PMC), comprising 30 for training, 30 for validation, and 49 for testing. These 109 documents were drawn from a set of 500 CoDiet documents undergoing full human NER annotation; they correspond to the subset that had been completely annotated and adjudicated by two human experts at the time of RECoDe development, while the remaining documents were still in adjudication at that point. The 30/30/49 split was performed at the document (PMCID) level by random assignment, so that no document appears in more than one split, avoiding leakage of co-occurring entity pairs between splits. For our experiments, we rely exclusively on the gold-standard named entity recognition (NER) annotations provided in the original corpus for the foodRelated, dietMethod, diseasePhenotype, geneSNP, proteinEnzyme, metabolite, and microbiome categories of annotations. The details of the annotation process and guidelines can be found in the CoDiet corpus paper (Lain *et al.* 2025).

**Figure 1:**
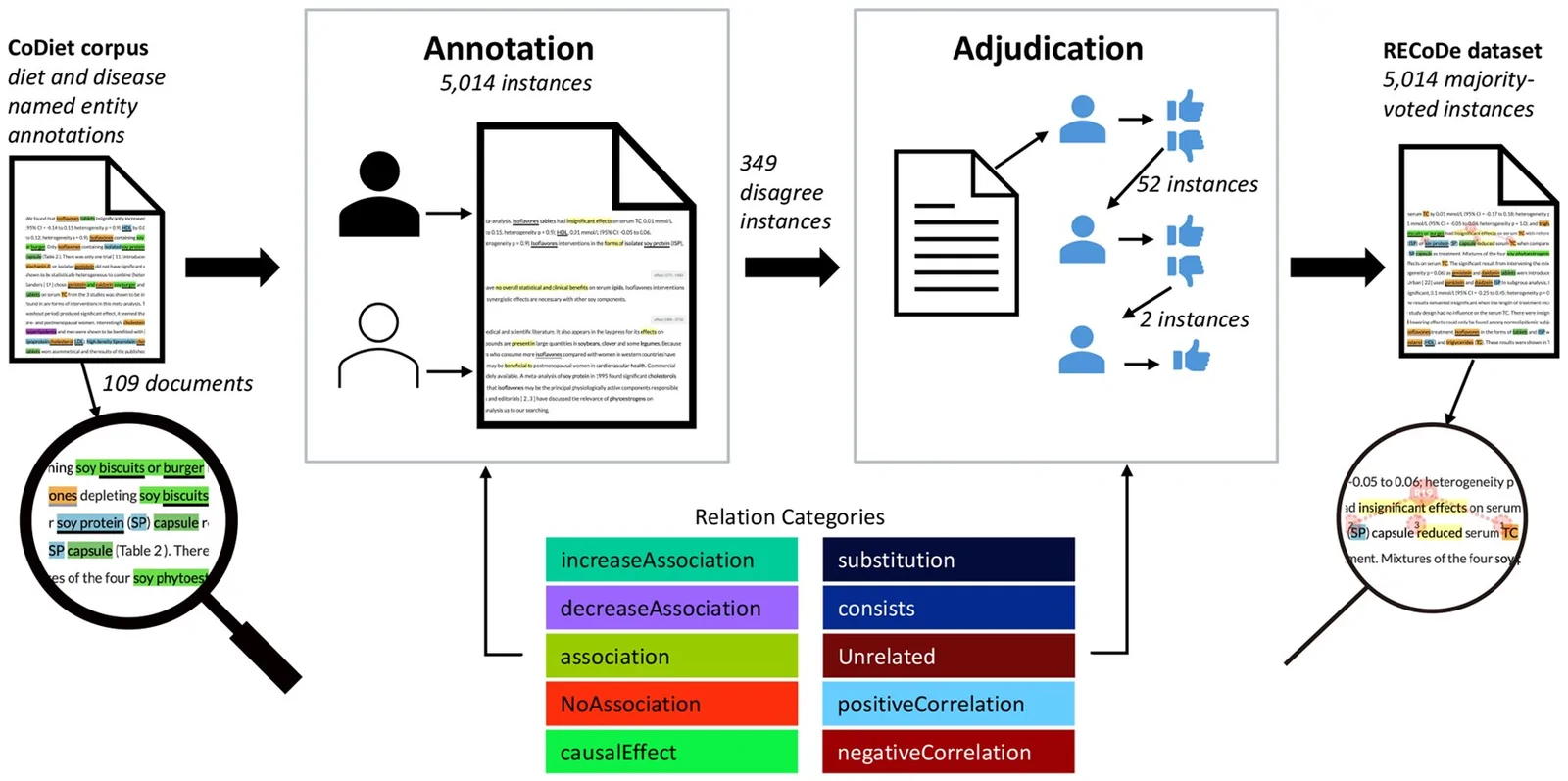
Overview of the annotation and adjudication process in the CoDiet corpus. A total of 5014 majority-voted instances were included, with 349 disagreements adjudicated across 109 documents.

RECoDe annotation was done in two phases: an annotation (two steps) and adjudication period (see Fig. 1). In the first step of the annotation period, one annotator was instructed to first select as many candidate relation pairs as possible across each entire document. Across the 109 documents, a total of 5014 relation instances were selected. In the second step, two annotators then independently annotated these 5014 potential relations, achieving an inter-annotator agreement of Cohen’s κ=0.919 (Cohen 1960) computed at the full 10-class granularity using sklearn.metrics.cohen_kappa_score. In the subsequent adjudication period, we ensured that all instances were finalised through majority voting. Since the task involves 10 relation classes, annotators occasionally selected different labels for the same instance. For such cases, additional annotators were involved to resolve the disagreements. Specifically, out of 5014 instances, 349 cases required adjudication. The first adjudicator looked at 349 disagreements, leaving 52 unresolved. A second adjudicator addressed these 52, leaving 2 remaining cases, which were finally adjudicated by a third independent person. In total, each relation included in the final dataset has been verified by 2 individuals. The test set is hosted on Codabench (https://www.codabench.org/competitions/14960/) to allow benchmarking.

Table 1 compares the distribution of relation types in major biomedical RE datasets. Traditional corpora (e.g. AIMed, ADE, DDI, N2C2, RENET2) are dominated by a small number of relation categories, often focusing on drug–adverse event or protein–protein interactions, and typically lack explicit negative relations. BioRED extends coverage to correlations, but does not capture dietary knowledge. In contrast, RECoDe covers a diverse set of relations, including *association* (19.8%), *causalEffect* (11.1%), *increase/decrease association* (25.8%), and explicit negatives such as *NoAssociation* (6.6%) and *Unrelated* (14.4%).

**Table 1:** Relation types in biomedical datasets.

Dataset	incrAssoc	decrAssoc	assoc	causal	subst	consist	posCorr	negCorr	noAssoc	Unrelated	Total
AIMed (Bunescu *et al.* 2005)	–	–	100%	–	–	–	–	–	–	–	1101
BioInfer (Pyysalo *et al.* 2007)	–	–	19%	–	–	21%	54%	6%	–	–	2662
EMU (Doughty *et al.* 2011)	–	–	100%	–	–	–	–	–	–	–	179
ADE (Gurulingappa *et al.* 2012)	–	–	4%	96%	–	–	–	–	–	–	7100
DDI (Herrero-Zazo *et al.* 2013)	–	–	59%	41%	–	–	–	–	–	–	5028
N-ary (Peng *et al.* 2017)	–	–	100%	–	–	–	–	–	–	–	3462
N2C2 (Henry *et al.* 2020)	–	–	97%	3%	–	–	–	–	–	–	59 810
DrugProt (Miranda *et al.* 2021)	17%	29%	54%	–	–	–	–	–	–	–	24 526
RENET2 (Su *et al.* 2021)	–	–	100%	–	–	–	–	–	–	–	51 642
BioRED (Luo *et al.* 2022)	–	–	55%	–	–	–	27%	18%	–	–	6503
DIMB-RE (Hong *et al.* 2025)	34%	20%	7%	23%	–	5%	7%	4%	–	–	4206
**RECoDe**	12.1%	13.7%	19.8%	11.1%	0.4%	16.0%	3.4%	2.4%	6.6%	14.4%	5014

See Table S1 for type definitions, Table S2 for examples, and Table S4 for the per-split breakdown. Negative relations are further categorized into NoAssociation and Unrelated. A “-” indicates the absence of a relation type in this dataset.

Detailed statistics of entity pair distributions across the train, validation, and test splits are provided in Figs. S1 and S2. These results confirm that RECoDe covers a broad range of diet/food, disease/phenotype, and biomedical entity combinations.

### 2.2 Experiments

The primary aim of our experiments is to identify the most effective and practically feasible approaches for RE within realistic academic resource constraints. Accordingly, we evaluate both supervised and generative approaches for the RECoDe RE task. For the supervised setting, we employ traditional BERT-like models trained using standard supervised learning on our annotated training dataset. In contrast, the generative setting explores recent advances in LLMs that can infer relations through prompting rather than parameter fine-tuning. Detailed descriptions of all evaluated models and evaluation protocols, including architecture, parameter sizes, deployment configurations, and performance metrics, are provided in the Models section of the Supplementary Materials.

### 2.3 Prompt and inference strategy

All prompt tuning experiments were conducted using the training set, which we regard as a form of supervised learning aimed at improving generalisability.

**Figure 2:**
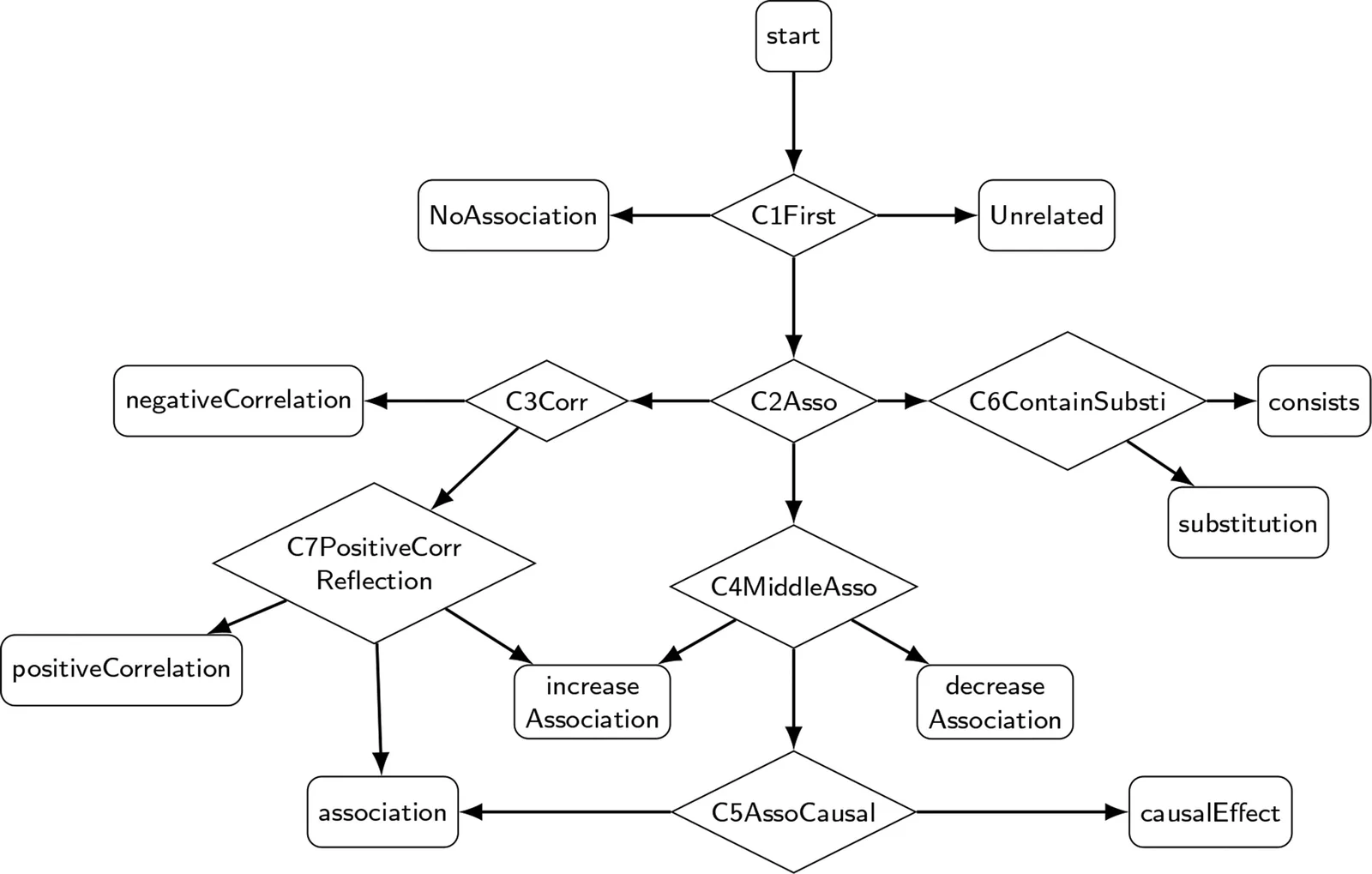
Decision flow of predict: staged classifiers (C1–C7) and branching on majority_pred.

Firstly, we applied a prompting approach using a set of well-established techniques, including role-play prompting (Tamoyan *et al.* 2024), guideline-based prompting, and example-augmented prompting, which have shown effectiveness in biomedical QA tasks (Nori *et al.* 2023). We propose a decision-flow framework, illustrated in Fig. 2.

The motivation arises from the observation that most local LLMs struggle with limited context length. To address this, we designed a multi-stage decision process inspired by decision trees, with a structured workflow resembling recent agentic workflow reasoning strategies (Ridnik *et al.* 2024). In the decision-making process, the model first distinguishes between positive relations and negative cases (*NoAssociation* and *Unrelated*). We found that providing more fine-grained definitions of *NoAssociation* and *Unrelated* improved performance compared to binary classification.

If the input is classified as a positive relation, the next stage divides it into three categories: *correlation*, *association*, and *contains*. Correlations are further separated into *positiveCorrelation* and *negativeCorrelation*. Associations are classified into *increaseAssociation* and *decreaseAssociation*. Finally, the *contains* branch is divided into *consists* and *substitution*. All prompts are presented in the Prompts section of the Supplementary Materials.

Each prompt begins with a system role-play instruction adapting LLMs to domain-specific tasks. And we provide detailed explanations of the answer classes. We further include guidelines refined through human trials on the training dataset, highlighting notable “dos and don’ts” such as explicitly stating that NoAssociation corresponds to “no effect”. To handle cases that are difficult to describe with textual guidelines alone, we add clear and distinguishable examples. Finally, we incorporate fine-grained instructions such as “Answer (A) Yes ONLY IF…” to enforce precise decision rules.

We also applied a specialised reflection step (Renze and Guven 2024) for the *positiveCorrelation* class, which is designed to allow the LLM to revise its initial prediction particularly for challenging or ambiguous cases. During initial trials with the training set, we observed that LLMs frequently confused *positiveCorrelation* with *increaseAssociation* or *association*.

### 2.4 Contextual Co-occurrence Summarisation

We define the concept of Contextual Co-occurrence Summarisation (CoCoS) as a means to combine the evidence found across different documents for the same entity-pair. Multiple extracted relations for the same entity pair within a document are aggregated into a single document-level relation using the procedure described in Algorithm 1.

Algorithm 1 CoCoS: Within-document relation aggregation using a rule-based approach.

**Require:** Sentence-level relation labels *R* for a given $\left(\mathrm{doc},e_{1},e_{2}\right)$ pair

**Ensure:** Aggregated document-level relation label $r^{*}$

1: Remove instances labelled *Unrelated* from *R*

2: Count occurrences of *positiveCorrelation*, *negativeCorrelation*, *association*, *increaseAssociation*, *decreaseAssociation*, *causalEffect*, *consists*, *substitution*, and *NoAssociation*, denoted as $\mathrm{cor}r_{\mathrm{pos}}$, $\mathrm{cor}r_{\mathrm{neg}}$, $\mathrm{asso}c_{\mathrm{neu}}$, $\mathrm{asso}c_{\mathrm{inc}}$, $\mathrm{asso}c_{\mathrm{dec}}$, *causal*, *consist*, *subst*, and *noAssoc*, respectively.

3: $\mathrm{cor}r_{\mathrm{total}}\leftarrow \mathrm{cor}r_{\mathrm{pos}}+\mathrm{cor}r_{\mathrm{neg}}$

4: $\mathrm{asso}c_{\mathrm{total}}\leftarrow \mathrm{asso}c_{\mathrm{neu}}+\mathrm{asso}c_{\mathrm{inc}}+\mathrm{asso}c_{\mathrm{dec}}+\mathrm{causal}$

5: $\mathrm{consis}t_{\mathrm{total}}\leftarrow \mathrm{consist}+\mathrm{subst}$

6: $\mathrm{anyAssoc}\leftarrow \mathrm{cor}r_{\mathrm{total}}+\mathrm{asso}c_{\mathrm{total}}+\mathrm{consis}t_{\mathrm{total}}$

7: **if**  noAssoc>anyAssoc  **then** 

8:  **return** *NoAssociation*

9: **end if** 

10: Determine winning group *G* with maximum value among:

11:  correlation ($\mathrm{cor}r_{\mathrm{total}}$),

12:  association ($\mathrm{asso}c_{\mathrm{total}}$),

13:  consist/substitution ($\mathrm{consis}t_{\mathrm{total}}$)

14:  (tie-break priority: correlation > association >consist/substitution)

15: **if**  G= correlation **then** 

16:  **if**  $\mathrm{cor}r_{\mathrm{pos}}\geq 2\times \mathrm{cor}r_{\mathrm{neg}}$  **then** 

17:   **return** *positiveCorrelation*

18:  **else if**  $\mathrm{cor}r_{\mathrm{neg}}\geq 2\times \mathrm{cor}r_{\mathrm{pos}}$  **then** 

19:   **return** *negativeCorrelation*

20:  **else** 

21:   **return** *association*

22:  **end if** 

23: **end if** 

24: **if**  G= association **then** 

25:  **if**  $\mathrm{causal}>\mathrm{asso}c_{\mathrm{neu}}+\mathrm{asso}c_{\mathrm{inc}}+\mathrm{asso}c_{\mathrm{dec}}$  **then** 

26:   **return** *causalEffect*

27:  **end if** 

28:  **if**  $\mathrm{asso}c_{\mathrm{inc}}\geq 2\times \mathrm{asso}c_{\mathrm{dec}}$  **then** 

29:   **return** *increaseAssociation*

30:  **else if**  $\mathrm{asso}c_{\mathrm{dec}}\geq 2\times \mathrm{asso}c_{\mathrm{inc}}$  **then** 

31:   **return** *decreaseAssociation*

32:  **else** 

33:   **return** *association*

34:  **end if** 

35: **end if** 

36: **if**  G= consist/substitution **then** 

37:  **if**  consist≥subst  **then** 

38:   **return** *consists*

39:  **else** 

40:   **return** *substitution*

41:  **end if** 

42: **end if** 

These document-level summarised extracted relations are then aggregated into two scores for each entity pair. The first is the Association Support (AS) score indicating the evidence for an association in general (Equation 1), and the second is an Effect Estimate (EE) score that indicates the direction (direct, inverse) of the association (Equation 2) using terminology from Fig. 2 to indicate elements:


(1) $$AS=\frac{N_{\text{assoc}}}{N_{\text{assoc}}+N_{\text{no}}}$$

(2) $$EE=\frac{N_{\text{pos}}-N_{\text{neg}}}{N_{\text{assoc}}}$$

where $N_{\text{pos}}$, $N_{\text{neg}}$, and $N_{\text{neu}}$ denote the number of documents whose aggregated relation corresponds to positive evidence (*increaseAssociation*, *positiveCorrelation*, or *consists*), negative evidence (*decreaseAssociation*, *negativeCorrelation*, or *substitution*), and neutral evidence (*causalEffect* or *association*), respectively. $N_{\text{assoc}}=N_{\text{pos}}+N_{\text{neg}}+N_{\text{neu}}$, and $N_{\text{no}}$ denotes the number of documents labelled as *NoAssociation*. All counts refer to document-level aggregated relations (with *Unrelated* excluded).

Equation 1 (AS) signifies the grouping of direct, indeterminate, and inverse relations over total number of relations (including no association but excluding unrelated) to give a score limited between 0 and 1. Equation 2 (EE) gives the direction of the combined evidence (direct associations minus inverse associations over the total number of associations) with the score ranging from -1 to 1. Here 0 indicates there is equal evidence for either side, and -1/+1 indicates all evidence is inverse/direct, respectively.

As an illustrative application of the CoCoS pipeline, we curated a subset of relations centered on key dietary factors from the application of the RE inference on the CoDiet-Electrum corpus Lain *et al.* (2025) (n = 4445 documents). Specifically, we focus on relations between food-related entity mentions [e.g. sugar-sweetened beverage and high-fat diet (HFD), including Western diet] and disease/phenotype mentions (e.g. insulin resistance/sensitivity/tolerance, type-2 diabetes, obesity/obese/overweight, and liver inflammation/injury). Furthermore, we examine how both groups are associated with commonly reported proteins [high-density lipoprotein (HDL) cholesterol, low-density lipoprotein (LDL) cholesterol, and alanineaminotransferase(ALT)] and microbiota (*Firmicutes* and *Bacteroidetes*).

## 3 Results

### 3.1 Model selection (validation dataset)

We evaluated 14 LMs, each with 2–3 different strategies, using the training data, and selected the best-performing models for final test set evaluation. To ensure a fair and unbiased comparison, model selection was performed exclusively on the validation set based on the highest multi-class F1 score within each model architecture (BERT, LLM), training domain (general, biomedical), and training strategy (zero-shot, hierarchical, reflection).

See the Supplementary Materials (Validation Results, Table S3) for the full results table. The top two models for the fine-tuned BERT architecture were *BioLinkBERT* (0.64) and *BioBERT* (0.64); for general-purpose LLMs, *gpt-oss-20B* (0.65) and *Llama-3.3-70B* (0.63); and for biomedical domain-adapted LLMs, *Meerkat-70B* (0.65) and *MedGemma-27B* (0.62). Hierarchical prompting contributes a negligible (0.1%) average increase in macro-F1 over zero-shot across models; adding the reflection step (hierarchical+reflection) increases macro-F1 by 1.6% on average over hierarchical alone. *MedGemma-27B* is the exception and performs best at zero-shot. At the per-class level (Table S5), BERT-family models remain strong on common classes, with *gpt-oss-20B* providing additional gains on rare classes (e.g. *substitution*). The test set was reserved only for final performance assessment to identify the most suitable modelling paradigm for diet–disease–bioentity RE and was not used for model training, tuning, or selection.

### 3.2 Multi-class classification (test dataset)

In our evaluation, *gpt-oss-20B* consistently outperformed all other models across accuracy, macro F1, precision, and recall on the test set, as shown in Table 2. It achieved an accuracy of 0.66, a macro-F1 of 0.61, a precision of 0.66, and a recall of 0.59, marking the overall best performance among all compared models. The 20B-parameter model outperformed both biomedical-specific supervised models *BioLinkBERT* (accuracy 0.51, macro-F1 0.48) and *BioBERT* (accuracy 0.50, macro-F1 0.47), as well as larger domain-adapted generative models *MedGemma-27B* (accuracy 0.57, macro-F1 0.56) and *Meerkat-70B* (accuracy 0.55, macro-F1 0.53).

**Table 2:** Multi-class classification results for the test set.

Model name	Type	Accuracy	F1 Score	Precision	Recall
**BioBERT**	normal	0.4974	0.4727	0.5069	0.5131
**BioLinkBERT**	normal	0.5125	0.4844	0.5074	0.5550
**Meerkat-70B**	H + R	0.5521	0.5338	0.5483	0.5655
**MedGemma-27B**	zero-shot	0.5677	0.5644	0.6277	0.5563
**Llama-3.3-70B**	H + R	0.5776	0.5499	0.5846	0.5473
**gpt-oss-20B**	H + R	**0.6612**	**0.6068**	**0.6578**	**0.5930**

Top result for each metric is given in bold font, and second best is underlined. F1, precision and recall are reported as macro. Type indicates the strategy used to optimise the model, see Supplementary Materials (Validation Results, Table S3; H + R = hierarchical prompt + reflection).

### 3.3 Binary classification result (test dataset)

From an information extraction perspective, a key objective is to reliably distinguish whether a given entity pair should be extracted for downstream use or disregarded. To this end, we reformulated the task as a binary classification problem, grouping *Unrelated* and *NoAssociation* into the negative class and all remaining relation types into the positive class. In the binary classification setting, *gpt-oss-20B* achieved the strongest overall performance (F1), as shown in Table 3. Specifically, its accuracy was 0.85, binary F1 0.89, precision 0.90, and recall 0.88. The next best model in terms of F1, *Llama-3.3-70B*, obtained lower general scores (accuracy 0.81, F1 0.87, precision 0.82) but with a larger recall of 0.94. The only metric for which *gpt-oss-20B* did not achieve the best performance was the binary recall for which *BioBERT* and *BioLinkBERT* both achieved 0.98, and where *MedGemma-27B* was the best LLM (0.95).

**Table 3:** Binary classification results for the test set.

Model name	Type	Accuracy	F1 Score	Precision	Recall
**BioBERT**	normal	0.7598	0.8493	0.7484	**0.9815**
**BioLinkBERT**	normal	0.7626	0.8506	0.7513	0.9802
**Meerkat-70B**	H + R	0.7900	0.8542	0.8191	0.8925
**MedGemma-27B**	zero-shot	0.8084	0.8718	0.8090	0.9452
**Llama-3.3-70B**	H + R	0.8145	0.8745	0.8198	0.9370
**gpt-oss-20B**	H + R	**0.8532**	**0.8924**	**0.9021**	0.8830

Top result for each metric is given in bold font, and second best is underlined. F1, precision and recall are reported as per the positive class. Type indicates the strategy used to optimise the model (H + R = hierarchical prompt + reflection).

### 3.4 Multi-document relation summarisation

Integrating the evidence across multiple documents and quantifying the associations between food-related items and health indicators (both disease phenotypes and bioentities) was done by calculating two scores: the Association Support (AS) score that reflects the overall evidence for any relationship, and the Effect Estimate (EE) score that indicates the direction of that relationship as either direct or inverse.

This was visualised as a directed graph in Fig. S3, with the corresponding CoCoS scores for selected relations summarised in Table 4. The strongest evidence was found for HFD impacting on LDL-cholesterol with an EE score of 0.75, indicating that at least 75% of 28 documents (28/29 documents report any type of association, AS score = 0.97) indicate it is a direct relationship opposed to any other type. The relationship most prevalent in the corpus is HFD with obesity/obese with an EE of 0.71/0.65, with an AS of 0.96/1.00. The microbiota *Firmicutes* (direct) and *Bacteroidetes* (inverse) also have consistent EE scores with HFD (0.71/-0.73), obese (0.52/-0.58), and obesity (0.33/-0.50), but opposite with overweight (-0.13/0.29) with the low scores indicating the conflicting evidence for overweight opposed to obese groups.

**Table 4:** CoCos example across 4445 documents from the CoDiet corpus (Lain *et al.* 2025) showing all scores for relations found in at least 10 documents.

Entity 1	Entity 2	#Docs	AS score	EE score
HFD	LDL-C	29	0.966	0.750
HFD	Bacteroidetes	60	0.917	−0.727
HFD	Firmicutes	79	0.975	0.714
HFD	Obesity	169	0.964	0.706
HFD	ALT	51	0.922	0.681
HFD	Insulin resistance	59	0.966	0.667
Liver injury	ALT	13	0.923	0.667
HFD	Obese	46	1.000	0.652
Insulin resistance	HDL-C	10	1.000	−0.600
Obese	Bacteroidetes	29	0.828	−0.583
Obese	Firmicutes	28	0.893	0.520
Obesity	Bacteroidetes	26	0.923	−0.500
Overweight	HDL-C	13	0.923	−0.417
HFD	HDL-C	65	0.969	0.413
HFD	Insulin sensitivity	25	0.920	−0.391
HFD	T2DM	43	0.977	0.381
Obesity	Firmicutes	32	0.938	0.333
Obesity	ALT	10	1.000	0.300
Obesity	HDL-C	21	0.857	−0.167
Obese	HDL-C	15	0.933	−0.143
Overweight	Firmicutes	10	0.800	−0.125

The illustration is shown in Fig. S3. Association Support (AS) score (Equation 1) indicates the evidence found for an association (0 to 1), and the Effect Estimate (EE) score (Equation 2) indicates the directionality (-1 to 1). Abbreviations: HFD = high-fat diet, T2DM = type-2 diabetes mellitus, HDLC = high-density lipoprotein cholesterol, LDLC = low-density lipoprotein cholesterol, ALT = alanine aminotransferase.

## 4 Discussion

We contributed a dedicated diet–disease-bioentity RE dataset covering 10 different relation types, including for the first time explicit negative examples. We evaluated both fine-tuned BERT- and LLM prompt-based models for their capabilities to capture complex relations between entities. We showed that generative LLMs, when guided with structured prompting strategies, hold strong potential for biomedical information extraction with an F1 close to 0.90 for binary classification and multi-class classification over 0.60 F1. Our evaluation reveals that generative models demonstrate significantly better generalisation compared to such supervised models. For instance, while the supervised *BioBERT* model dropped from a macro-F1 of 0.64 on the validation set to 0.48 on the test set, our top model (*gpt-oss-20B*) maintained stable performance, achieving 0.65 and 0.61 on validation and test, respectively. This substantial gap indicates that prompt-based generative models exhibit far greater robustness to domain shifts and unseen examples than traditional supervised architectures, which tend to overfit both the training distribution and the hyperparameter optimisation process that is tightly tuned to the validation set. For example, we demonstrated that *DiMB-RE* was heavily dependent on the gold-standard labels within their benchmark, as it did not generalise to new/out-of-context data here at an F1 of 0.29 (Table S3). While our best model achieved an F1 of 0.61 (multi-class) in an end-to-end scenario (machine annotations), *DiMB-RE* reported a drop in F1 from 0.81 to 0.45 when moving from human annotated entities as inputs for RE to machine annotations for in-context data (Hong *et al.* 2025). Hence, these traditional supervised architectures remain sensitive to training distributions and validation-tuned hyperparameters, making prompting-based generative models potentially more suitable as they can achieve high robustness and balanced precision–recall trade-offs.

In biomedical RE, most models identify relationships between entities at the sentence or paragraph level. However, real-world evidence in biomedical literature is often distributed across multiple documents. To enable cross-document reasoning and synthesis we introduced CoCoS, a post-processing framework that aggregates RE results for identical entity pairs observed across different contexts. Unlike previous biomedical KG construction efforts such as PubMedKG (Xu *et al.* 2020), which primarily aggregate entities based on simple co-occurrence, our approach aggregates evidence at the document level to enable an automatic systematic review of diet–disease relationships along with bio-entities. Specifically, CoCoS is designed to summarise diverse and sometimes conflicting findings reported across multiple studies, where multiple independent RE outputs are contextualised and merged into a coherent summary that reflects the overall scientific consensus or disagreement. CoCoS effectively summarised several relations that are well-known with the correct effect estimate (sign) to demonstrate its potential for evidence synthesis. For example the link between obesity/obese (disease/disease group, respectively, were not normalised nor combined into a single entity to demonstrate CoCoS finds the same relations for both) and the high-fat diet with *Firmicutes* (direct) and *Bacteroidetes* (inverse). These relations reflect the association between obesity and the *Firmicutes*-to-*Bacteroidetes* ratio (Magne *et al.* 2020). Also, the opposite signs of CoCoS of LDL-C (bad) and HDL-C (good) with different diseases and phenotypes is also consistent with the current body of knowledge (Klop *et al.* 2013). On the other hand, CoCoS did not find evidence beyond “*association*” (1 document) between sugar-sweetened beverages and T2DM, while this is reported in the Burden of Proof (BoP) (Zheng *et al.* 2022) resource as a weak direct effect. We validated CoCoS against well-known diet–disease relationships first, since replicating established meta-analytic findings is a prerequisite for subsequent application to novel hypotheses. This could leave some limitations of the current methodology unobserved, which we expect to discover in ongoing and future work.

However, the BoP only considers randomised controlled trials and relies on manual effort to extract data and perform the meta-analyses. Hence, only 88 of the 177 BoP entries relate to modifiable dietary or cardiometabolic risks, of which 48 relate to outcomes linked to metabolic syndrome. In contrast, our approach is fully computational and aims to bring together evidence across all study methodologies. In future work, we aim to release an extended version of CoCoS that integrates document-level weighting based on study methodology classification aligned to the hierarchy of evidence. This will bridge the gap between the fast computational approaches for systematic analysis and the time-intensive human systematic review process and generate testable hypotheses for future meta-analyses. Adding in the population sample size and statistical significance reported in each document into the score could further increase the accuracy and alignment with meta-analyses that do incorporate these data.

In summary, our results demonstrate on a newly created corpus that generative LLMs outperform supervised models for binary discrimination tasks, where the goal is to identify whether a relation exists between two entities, and extend well to multi-class RE. These models can be used to pre-select relevant bits of information for human review at a fraction of the time that it would take a human reader. Further improving these models will make their use alongside a human researcher in a systematic review a not-so-distant possibility.

## Author contributions

Donghee Choi (Methodology, Software, Validation, Formal analysis, Investigation, Data Curation, Writing—original draft, Writing—review & editing, Visualization, Supervision), Yajie Gu (Methodology, Software, Investigation, Writing—review & editing, Visualization), Kai Qi Zong (Investigation, Data Curation), Antoine D. Lain (Validation, Data Curation), Dimitrios Zaikis (Data Curation), Thomas Rowlands (Software), Marek Rei (Conceptualization, Resources, Funding acquisition), The CoDiet Consortium (Funding acquisition), Tim Beck (Conceptualization, Supervision, Funding acquisition), and Joram M. Posma (Conceptualization, Methodology, Validation, Formal analysis, Resources, Data Curation, Writing—review & editing, Visualization, Supervision, Project administration, Funding acquisition)

## Supplementary Material

btag439_Supplementary_Data

## Data Availability

The code, models, and dataset are publicly available at https://github.com/omicsNLP/RECoDe. The data are hosted on Zenodo at https://doi.org/10.5281/zenodo.19050553/. The code was archived on Zenodo at https://doi.org/10.5281/zenodo.20613883. The benchmark is hosted on Codabench at https://www.codabench.org/competitions/14960/.

## References

[btag439-B1] Bunescu R , Ge R, Kate RJ et al Comparative experiments on learning information extractors for proteins and their interactions. Artif Intell Med 2005;33:139–55. 15811782 10.1016/j.artmed.2004.07.016

[btag439-B2] Cohen J. A coefficient of agreement for nominal scales. Educ Psychol Meas 1960;20:37–46.

[btag439-B3] Dagbasi A , Fuller A, Hanyaloglu AC et al The role of nutrient sensing dysregulation in anorexia of ageing: the little we know and the much we don’t. Appetite 2024;203:107718. 39423861 10.1016/j.appet.2024.107718

[btag439-B4] Devlin J , Chang M-W, Lee K et al Bert: pre-training of deep bidirectional transformers for language understanding. In: *Proceedings of the 2019 Conference of the North American Chapter of the Association for Computational Linguistics: Human Language Technologies, Volume 1 (Long and Short Papers)*, pp.4171–86, 2019.

[btag439-B5] Doughty E , Kertesz-Farkas A, Bodenreider O et al Toward an automatic method for extracting cancer-and other disease-related point mutations from the biomedical literature. Bioinformatics 2011;27:408–15. 21138947 10.1093/bioinformatics/btq667 PMC3031038

[btag439-B6] Geleta D , Nikolov A, Edwards G et al Biological insights knowledge graph: an integrated knowledge graph to support drug development. In: *WSDM 2022: International Conference on Web Search and Data Mining*. Biorxiv, 2021–10, 2021. 10.1101/2021.10.28.466262

[btag439-B7] Gurulingappa H , Rajput AM, Roberts A et al Development of a benchmark corpus to support the automatic extraction of drug-related adverse effects from medical case reports. J Biomed Inform 2012;45:885–92. 22554702 10.1016/j.jbi.2012.04.008

[btag439-B8] Henry S , Buchan K, Filannino M et al 2018 n2c2 shared task on adverse drug events and medication extraction in electronic health records. J Am Med Inform Assoc 2020;27:3–12. 31584655 10.1093/jamia/ocz166 PMC7489085

[btag439-B9] Herrero-Zazo M , Segura-Bedmar I, Martínez P et al The DDI corpus: an annotated corpus with pharmacological substances and drug–drug interactions. J Biomed Inform 2013;46:914–20. 23906817 10.1016/j.jbi.2013.07.011

[btag439-B10] Hong G , Hindle V, Veasley NM et al DiMB-RE: mining the scientific literature for diet-microbiome associations. J Am Med Inform Assoc 2025;32:998–1006. 40152137 10.1093/jamia/ocaf054 PMC12089768

[btag439-B11] Khraisha Q , Put S, Kappenberg J et al Can large language models replace humans in systematic reviews? Evaluating GPT-4’s efficacy in screening and extracting data from peer-reviewed and grey literature in multiple languages. Res Synth Methods 2024;15:616–26. 38484744 10.1002/jrsm.1715

[btag439-B12] Klop B , Elte J, Cabezas M. Dyslipidemia in obesity: mechanisms and potential targets. Nutrients 2013;5:1218–40. 10.3390/nu5041218 23584084 PMC3705344

[btag439-B13] Lai P-T , Wei C-H, Luo L et al BioREx: improving biomedical relation extraction by leveraging heterogeneous datasets. J Biomed Inform 2023;146:104487. 37673376 10.1016/j.jbi.2023.104487

[btag439-B14] Lain AD , Go S, Mahmud A et al Codiet corpus. bioRxiv, 2025. 10.1101/2025.09.04.673740

[btag439-B15] Lee M , Lee K, Yu N et al ChimerDB 3.0: an enhanced database for fusion genes from cancer transcriptome and literature data mining. Nucleic Acids Res 2017;45:D784–9. 27899563 10.1093/nar/gkw1083 PMC5210563

[btag439-B16] Lichtenstein AH , Yetley EA, Lau J. Application of systematic review methodology to the field of nutrition. J Nutr 2008;138:2297–306. 19022948 10.3945/jn.108.097154 PMC3415860

[btag439-B17] Lim S , Lee K, Kang J. Drug drug interaction extraction from the literature using a recursive neural network. PLoS One 2018;13:e0190926. 29373599 10.1371/journal.pone.0190926 PMC5786304

[btag439-B18] Lovegrove JA. Dietary fats and cardiometabolic health–from public health to personalised nutrition: ‘one for all’ and ‘all for one’. Nutr Bull 2025;50:132–41. 39833097 10.1111/nbu.12722 PMC11815623

[btag439-B19] Luo L , Lai P-T, Wei C-H et al BioRED: a rich biomedical relation extraction dataset. Brief Bioinform 2022;23:bbac282. 35849818 10.1093/bib/bbac282 PMC9487702

[btag439-B20] Magne F , Gotteland M, Gauthier L et al The firmicutes/bacteroidetes ratio: a relevant marker of gut dysbiosis in obese patients? Nutrients 2020;12:1474. 10.3390/nu12051474 32438689 PMC7285218

[btag439-B21] Miranda A , Mehryary F, Luoma J et al Overview of drugprot biocreative vii track: quality evaluation and large scale text mining of drug-gene/protein relations. In *Proceedings of the Seventh BioCreative Challenge Evaluation Workshop*, pp.11–21, 2021.

[btag439-B22] Nori H , Lee YT, Zhang S et al Can generalist foundation models outcompete special-purpose tuning? Case study in medicine. Medicine (Baltimore) 2023;84:77–3.

[btag439-B23] Oude Griep LM , Chekmeneva E, Van Horn L et al A metabolome wide association study of fruit and vegetable consumption and associations with cardiovascular disease risk factors: the international study of macro-/micronutrients and blood pressure (INTERMAP) study. J Nutr 2025;155:122–31. 39536968 10.1016/j.tjnut.2024.11.004 PMC11795696

[btag439-B24] Peng N , Poon H, Quirk C et al Cross-sentence N-ary relation extraction with graph LSTMs. Trans Assoc Comput Linguist 2017;5:101–15.

[btag439-B25] Pugh JE , Petropoulou K, Vasconcelos JC et al Increase in colonic propionate as a method of preventing weight gain over 12 months in adults aged 20–40 years (iPREVENT): a multi-Centre, double-blind, randomised, parallel-group trial. EClinicalMedicine 2024;76:102844. 39391015 10.1016/j.eclinm.2024.102844 PMC11466568

[btag439-B26] Pyysalo S , Ginter F, Heimonen J et al Bioinfer: a corpus for information extraction in the biomedical domain. BMC Bioinformatics 2007;8:50–24. 17291334 10.1186/1471-2105-8-50 PMC1808065

[btag439-B27] Renze M , Guven E. Self-reflection in LLM agents: effects on problem-solving performance. In: *2nd International Conference on Foundation and Large Language Models (FLLM)*, pp.516–25, 2024. 10.1109/FLLM63129.2024.10852426

[btag439-B28] Ridnik T , Kredo D, Friedman I. Code generation with alphacodium: from prompt engineering to flow engineering. arXiv preprint arXiv: 2401.08500, 2024, preprint: not peer reviewed.

[btag439-B29] Su J , Wu Y, Ting H-F et al RENET2: high-performance full-text gene–disease relation extraction with iterative training data expansion. NAR Genom Bioinform 2021;3:lqab062. 34235433 10.1093/nargab/lqab062 PMC8256824

[btag439-B30] Tamoyan H , Schuff H, Gurevych I. LLM roleplay: simulating human-chatbot interaction. In: *Proceedings of the Third Workshop on Social Influence in Conversations (SICon 2025)*, 2024.

[btag439-B31] Xu J , Kim S, Song M et al Building a pubmed knowledge graph. Sci Data 2020;7:205. 32591513 10.1038/s41597-020-0543-2 PMC7320186

[btag439-B32] Yoon W , Jackson R, Ford E et al Biomedical ner for the enterprise with distillated bern2 and the kazu framework. In: *Proceedings of the 2022 Conference on Empirical Methods in Natural Language Processing: Industry Track*, pp. 619–26, 2022.

[btag439-B33] Zheng P , Afshin A, Biryukov S et al The burden of proof studies: assessing the evidence of risk. Nat Med 2022;28:2038–44. 10.1038/s41591-022-01973-2 36216935 PMC9556298

