# Supplementary material for: Relation extraction for diet, non-communicable disease and biomarker associations (RECoDe): a CoDiet study
Source: Bioinformatics. 2026 Aug 21;42(Suppl 2):btag439. doi: 10.1093/bioinformatics/btag439 (PMC13501294; doi:10.1093/bioinformatics/btag439)
Supplement: Supplementary file 1 [file btag439_supplementary_data.pdf]

Relation Extraction for Diet, Non-Communicable Disease and Biomarker Associations (RECoDe): A CoDiet study - Supplementary Material

Donghee Choi,[1,2] Yajie Gu,[3] Kai Qi Zong,[1] Antoine D. Lain,[1] Dimitrios Zaikis,[4] Thomas Rowlands,[3] Marek Rei,[5] The CoDiet consortium,[6] Tim Beck[3] and Joram M. Posma[1,*]

[1]Section of Bioinformatics, Division of Systems Medicine, Department of Metabolism, Digestion and Reproduction, Imperial College London, W12 0NN, United Kingdom
[2]School of Computer Science and Engineering, Pusan National University, Busan, South Korea
[3]School of Medicine, Biodiscovery Institute, University of Nottingham, NG7 2RD, United Kingdom
[4]School of Informatics, Aristotle University of Thessaloniki, 57001, Greece
[5]Department of Computing, Imperial College London, SW7 2AZ, United Kingdom
[6]CoDiet Project, https://www.codiet.eu/

*Corresponding author. jmp111@ic.ac.uk

Type definitions

A total of 10 relation types were used for annotating the documents. Each annotator was given a document with the definition for each type and some examples. Table S1 shows the types and definitions, and Table S2 shows the examples for each type. The per-class distribution for the publicly released train and validation splits is reported in Table S4.

Models

We evaluate two categories of pretrained language models: **general-purpose LMs**, which serve as baselines across diverse NLP tasks, and **biomedical LMs**, which are specifically adapted to biomedical and clinical corpora. For further comparison, we apply the relation extraction pipeline from DiMB-RE on our diet-disease dataset to evaluate model performance, using a method originally developed for diet-microbiome relations.

In information extraction (IE) tasks, traditional BERT-like models trained with supervised learning remain the dominant paradigm, as they are effective for structured relation extraction and entity-level classification. However, due to their potential for broader generalisation, generative large language models have recently gained increasing attention. Accordingly, we evaluated both well-established BERT-based models and state-of-the-art generative models that could be locally deployed within our available GPU specifications.

For model deployment, we used HuggingFace (Wolf et al., 2019), vLLM (Kwon et al., 2023), and llama.cpp [1] implementations, and applied quantisation and optimisation techniques tailored to the NVIDIA L40S GPU.

Below, we summarise the models considered in each category. For the General-purpose LMs, we utilise the below models.

[1] https://github.com/ggml-org/llama.cpp

Table S1 Relation types defined in the RECoDe corpus.

Type	Definition
Unrelated	No linguistic relationship between co-occurring terms in the same sentence.
NoAssociation	Entities are linked but described as having no association.
Association	Entities are associated without any additional information on their type.
increaseAssociation	Entities are associated with specific indication of an increase. Other synonyms for increase are for example higher, activate, promote, enhances, improve. Focussed on a 'binary' entity's relation with a continuous one.
decreaseAssociation	Entities are associated with specific indication of a decrease. Other synonyms for decrease are lower, reduction, inhibit, worsen. Focussed on a 'binary' entity's relation with a continuous one.
positiveCorrelation	Associated entities indicated by a correlation (Pearson, Spearman, or others) that is positive (or direct). Focussed on two continuous entities.
negativeCorrelation	Associated entities indicated by a correlation (Pearson, Spearman, or others) that is negative (or inverse). Focussed on two continuous entities.
consists	An entity relationship where one entity can be seen as subset of the other.
substitution	An entity relationship where one entity is being replaced by the other.
causalEffect	Association between two entities that is described as causal from one entity with effect on the other.

Table S2 Examples for each relation types in the RECoDe corpus.

Type	Examples
Unrelated	"Homozygotes minor G allele at the rs6868989 had a significantly higher level of **HDL-C** and a lower level of fasting **glucose** than those with homozygous major A allele."
	"**Vascular resistances** increased in salt-sensitive and decreased in salt-resistant individuals after 5 days of high-salt diet without significant changes in **cardiac output**."
	"Vascular resistances increased in **salt-sensitive** and decreased in **salt-resistant individuals** after 5 days of high-salt diet without significant changes in cardiac output."
NoAssociation	"Common and low-frequency **variants in AGPAT6** do not significantly associate with **type 2 diabetes susceptibility**."
	"None of the eleven **AGPAT6 variants** were robustly associated with **type 2 diabetes** in the Danish case-control study."
	"Vascular resistances increased in **salt-sensitive** and decreased in salt-resistant individuals after 5 days of high-salt diet without significant changes in **cardiac output**."
	"Vascular resistances increased in salt-sensitive and decreased in **salt-resistant individuals** after 5 days of high-salt diet without significant changes in **cardiac output**."
association	"The minor G-allele of **rs17600159** showed nominal association with plasma **glucose** and insulin 2-h after an OGTT."
	"Mutations in AGPAT2, which is another gene involved in de novo triglyceride synthesis, is an accepted cause of **congenital generalized lipodystrophy** associated with **extreme insulin resistance** and early onset diabetes in humans."
	"Mutations in AGPAT2, which is another gene involved in de novo triglyceride synthesis, is an accepted cause of **congenital generalized lipodystrophy** associated with extreme insulin resistance and **early onset diabetes** in humans."
	"Vascular resistances increased in **salt-sensitive** and decreased in salt-resistant individuals after 5 days of **high-salt diet** without significant changes in cardiac output."
	"Vascular resistances increased in salt-sensitive and decreased in **salt-resistant individuals** after 5 days of **high-salt diet** without significant changes in cardiac output."
	"In Prevotella individuals, total DF and hemicellulose intake correlated positively with **adiposity** (evaluated as **body fat percentage**), while cellulose intake correlated with negatively with BMI."
increaseAssociation	"Homozygotes minor G allele at the **rs6868989** had a significantly higher level of **HDL-C** and a lower level of fasting glucose than those with homozygous major A allele."
	"**Vascular resistances** increased in **salt-sensitive** and decreased in salt-resistant individuals after 5 days of high-salt diet without significant changes in cardiac output."
	"Eating **LCHF diet** for four weeks induced a higher **LDL cholesterol** compared to control diet."
	"**Rabbit meat** is lean meat of high nutritive value, because it is rich in **essential amino acids**, polyunsaturated fatty acids (PUFA), vitamins, minerals."
	"**Rabbit meat** is lean meat of high nutritive value, because it is rich in essential amino acids, polyunsaturated fatty acids (PUFA), vitamins, **minerals**."
	"Some of these metabolites (Undecylenic acid, tocopheronic acid and corchorifatty acid F) have antioxidant activity that correlates with the fact that **BSDF** can improve **inflammation**."
decreaseAssociation	"Homozygotes minor G allele at the **rs6868989** had a significantly higher level of HDL-C and a lower level of fasting **glucose** than those with homozygous major A allele."
	"However, studies in normotensive humans revealed that an increased dietary **salt** intake (5–7 days of high-salt regimen) induces a profound reduction in vascular **nitric oxide** bioavailability"
	"**Vascular resistances** increased in salt-sensitive and decreased in **salt-resistant individuals** after 5 days of high-salt diet without significant changes in cardiac output."
positiveCorrelation	"In Prevotella individuals, **total DF** and hemicellulose intake correlated positively with **adiposity** (evaluated as body fat percentage), while cellulose intake correlated with negatively with BMI."
	"In Prevotella individuals, total DF and **hemicellulose** intake correlated positively with **adiposity** (evaluated as body fat percentage), while cellulose intake correlated with negatively with BMI."
	"Soluble fiber was negatively correlated with HDL cholesterol and weak positive correlations were observed between **cellulose** intake and serum **triglycerides** (TG) and between lignin consumption and serum ALT levels."
	"Soluble fiber was negatively correlated with HDL cholesterol and weak positive correlations were observed between cellulose intake and serum triglycerides (TG) and between **lignin** consumption and serum **ALT** levels."
negativeCorrelation	"In Prevotella individuals, total DF and hemicellulose intake correlated positively with adiposity (evaluated as body fat percentage), while **cellulose** intake correlated with negatively with **BMI**."
	"**Soluble fiber** was negatively correlated with **HDL cholesterol** and weak positive correlations were observed between cellulose intake and serum triglycerides (TG) and between lignin consumption and serum ALT levels."
consists	"**Oily fish** on the questionnaire included **mackerel**, Spanish mackerel, and saury."
	"**Intermittent fasting** (IF) is a dietary regime that refers to **short periods of intense energy restriction**."
	"The **diets** were formulated with **higher marine fishmeal/fish oil** and lower marine fishmeal/fish oil content by a commercial company."
	"The **diets** were formulated with higher marine fishmeal/fish oil and **lower marine fishmeal/fish oil** content by a commercial company."
	"Most sheep rations consist of **forages** with **high fibrous** content."
substitution	"**Carbohydrates** were replaced by **fat** (77% of daily energy intake (E%)), with a high proportion of saturated fatty acids (33E%)."
	"As a consequence of the European ban on antibiotic growth promoters (AGPs) and increased consumer awareness about the consumption of healthy and safer animal products, researchers and feed companies have encouraged to seek new **animal feeding** approaches to substitute **AGPs** and synthetic antioxidants."
causalEffect	"The first committed step in de novo triglyceride synthesis is the acylation of **glycerol-3-phosphate** leading to the formation of **lysophosphatidic acid**."
	"**Mutations in AGPAT2**, which is another gene involved in de novo triglyceride synthesis, is an accepted cause of **congenital generalized lipodystrophy** associated with extreme insulin resistance and early onset diabetes in humans."
	"**Myoglobin**, the sarcoplasmic heme protein, acts as the chief pigment accountable for the red color of the meat, is oxidized during storage, and will cause a **change in the meat color**."
	"**Vegetarian diets** caused significant **reduction in LDL-C** concentrations."

- **BERT-base** (Devlin et al., 2019): A standard Transformer-based model widely regarded as a strong baseline in general NLP.
- **Llama-3.1-8B** (Dubey et al., 2024): A medium-scale (8B parameters) model from the Llama family, capable of general text understanding and generation. We use the instruction version (Llama-3.1-8B-Instruct).
- **Llama-3.3-70B**: A large-scale model (70B parameters) with strong few-shot reasoning ability. We use the instruction version (Llama-3.3-70B-Instruct).
- **Qwen2.5-32B** (Yang et al., 2025): A multilingual model developed by Alibaba, achieving competitive results on general and reasoning tasks.
- **DeepSeek-R1-Qwen-32B** (Guo et al., 2025): A distilled variant of DeepSeek-R1 built upon Qwen2.5-32B, designed for improved efficiency–performance trade-offs (DeepSeek-R1-Distill-Qwen-32B).
- **DeepSeek-R1-Llama-70B**: A quantised and distilled variant of DeepSeek-R1 built upon Llama-3.3-70B, enabling memory-efficient inference while maintaining strong reasoning performance. We use llama.cpp to run this model with the q4_k_s version (DeepSeek-R1-Distill-Llama-70B-Q4_K_S).
- **gpt-oss-20B** (Agarwal et al., 2025): An open-weight reasoning model based on a mixture-of-experts Transformer architecture, trained through large-scale distillation and reinforcement learning, achieves strong general-purpose performance with notable strengths in reasoning and related tasks. We use vllm to utilise this model.

Also, we utilised biomedical LMs. We included a range of biomedical and clinical domain–adapted models to assess their performance in comparison with recent generative large language models that have been specialised for biomedical or medical text understanding.

- **BioBERT** (Lee et al., 2020): A domain-adapted BERT model pretrained on large-scale biomedical corpora, including PubMed abstracts and PMC articles.
- **PubMedBERT** (Gu et al., 2020): A biomedical-specific BERT trained exclusively on PubMed abstracts, yielding strong domain performance.
- **BioLinkBERT** (Yasunaga et al., 2022): A biomedical BERT variant aligned with knowledge graphs, effective for entity linking and relation extraction.
- **Meerkat-8B** (Kim et al., 2024): A lightweight biomedical LLM (8B parameters) specialised in clinical and biomedical text processing.
- **Meerkat-70B** (Kim et al., 2024): A large-scale biomedical LLM (70B parameters), designed for complex reasoning in biomedical and clinical domains.
- **MedGemma-27B** (Sellergren et al., 2025): An instruction-tuned biomedical model (27B parameters) from Google's Gemma (Team et al., 2024) family, tailored for clinical QA and instruction following.

Data splitting and evaluation metrics

For the multi-class classification setting, we report standard metrics including accuracy, and macro-averaged precision, recall, and F1-score. Macro averaging is used to account for label imbalance across classes. In addition, we also conduct a binary classification evaluation. Since one of our main goals is to construct a knowledge graph, it is essential to determine whether a given entity pair is related in any way. For this purpose, we group *NoAssociation* and *Unrelated* into the negative class, and all other relation types into the positive class. For this binary task, we again evaluate using accuracy and macro-averaged precision, recall, and F1-score.

Validation Results

Hierarchical Individual Expert Setting for BERT baselines.

To enable a fair comparison with the hierarchical prompting strategy used for generative LLMs, we also implemented a hierarchical classification variant for BERT-based models (Hierarchical - Individual Experts) in Table S3. In this setup, the original multi-class relation labels were decomposed into a sequence of stage-specific classification tasks corresponding to the decision flow shown in Figure 2. Separate classifiers ("individual experts") were trained for each stage using relabelled training data appropriate for that decision step. During inference on the validation and test sets, predictions were generated sequentially by applying the classifiers following the same hierarchical decision framework as the prompting pipeline. This experiment was designed to evaluate whether hierarchical decision decomposition itself improves performance independently of the generative reasoning capability of LLMs.

Multi-class classification on the validation set

The validation set results in Table S3 show that the performance varies considerably across architectures and inference strategies. We therefore selected the top two models in terms of macro F1 from each category (general LMs, biomedical LMs, and generative LLMs). These selected models were then carried forward to the independent test set evaluation. This design ensured a fair comparison between traditional supervised models and large generative LLMs under different prompting strategies, while preventing overfitting to a single inference style.

Our proposed *gpt-oss-20B* model with the *Hierarchical prompt + reflection* strategy demonstrated strong generalisation capability, achieving the highest macro F1-score (0.6731). This result indicates that prompt-based generative LLMs can capture underrepresented relation types more effectively, thereby overcoming the data distribution bias often observed in supervised learning. Nevertheless, the BERT-based supervised models also showed notable strengths. Despite being considerably smaller and computationally lighter, they achieved competitive accuracy and precision compared to large generative models. For instance, the *DiMB-RE* model obtained the highest overall accuracy (0.7238), outperforming our best-performing generative model in that metric (*gpt-oss-20B*, 0.6741).

The proposed *Hierarchical prompting* strategy proved particularly effective for generative LLMs, leading to consistent performance improvements across most models. In contrast, it resulted in performance degradation in all BERT-based models, suggesting that hierarchical decision-making is not a universal performance booster. Instead, it is especially beneficial for generative models when properly designed to mitigate context-length limitations and enhance reasoning depth through structured decomposition.

Domain adaptation benefits traditional supervised BERT models but not modern generative LLMs. In the BERT-based group,

Figure S1 Top 20 entity pair distributions across splits (train/validation/test). Each pie chart shows the percentage composition of the most frequent top pairs. The bottom-right panel shows the colour legend for entity-pair categories.

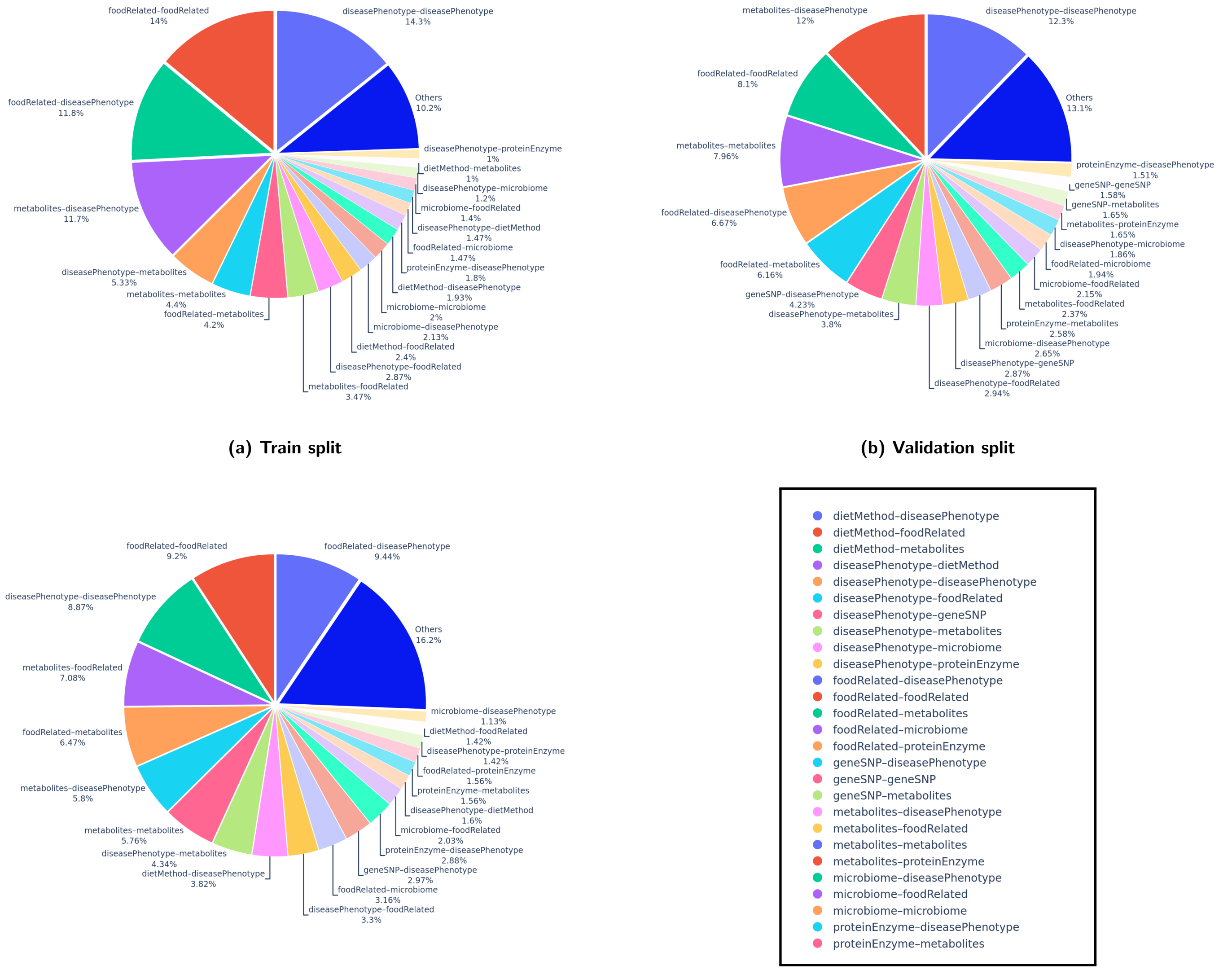

(a) Train split

(b) Validation split

(c) Test split

(d) Legend

domain-adapted biomedical LMs like BioBERT, PubMedBERT outperformed general models, indicating that domain adaptation remains advantageous for supervised learning with BERT-like LMs. In contrast, the general-purpose *gpt-oss-20B* significantly outperformed domain-specific biomedical LLMs such as *Meerkat-70B* and *MedGemma-27B*. This suggests that modern instruction-tuned generative models are less dependent on domain-specific corpora and rely more on their reasoning and instruction-following capabilities. Biomedical generative LLMs are often validated for question answering rather than information extraction, which may explain their relatively weaker performance in this task.

Finally, we observe consistent gains when applying our proposed reflection mechanism in generative LLMs. For the *gpt-oss-20B* model, both hierarchical prompting and the reflection step contributed to incremental improvements in F1 and recall, confirming that structured multi-stage reasoning and self-reflection enable more stable and accurate decision-making in complex relation extraction tasks.

Figure S2 Entity-pair category coverage. (a) train; (b) validation.

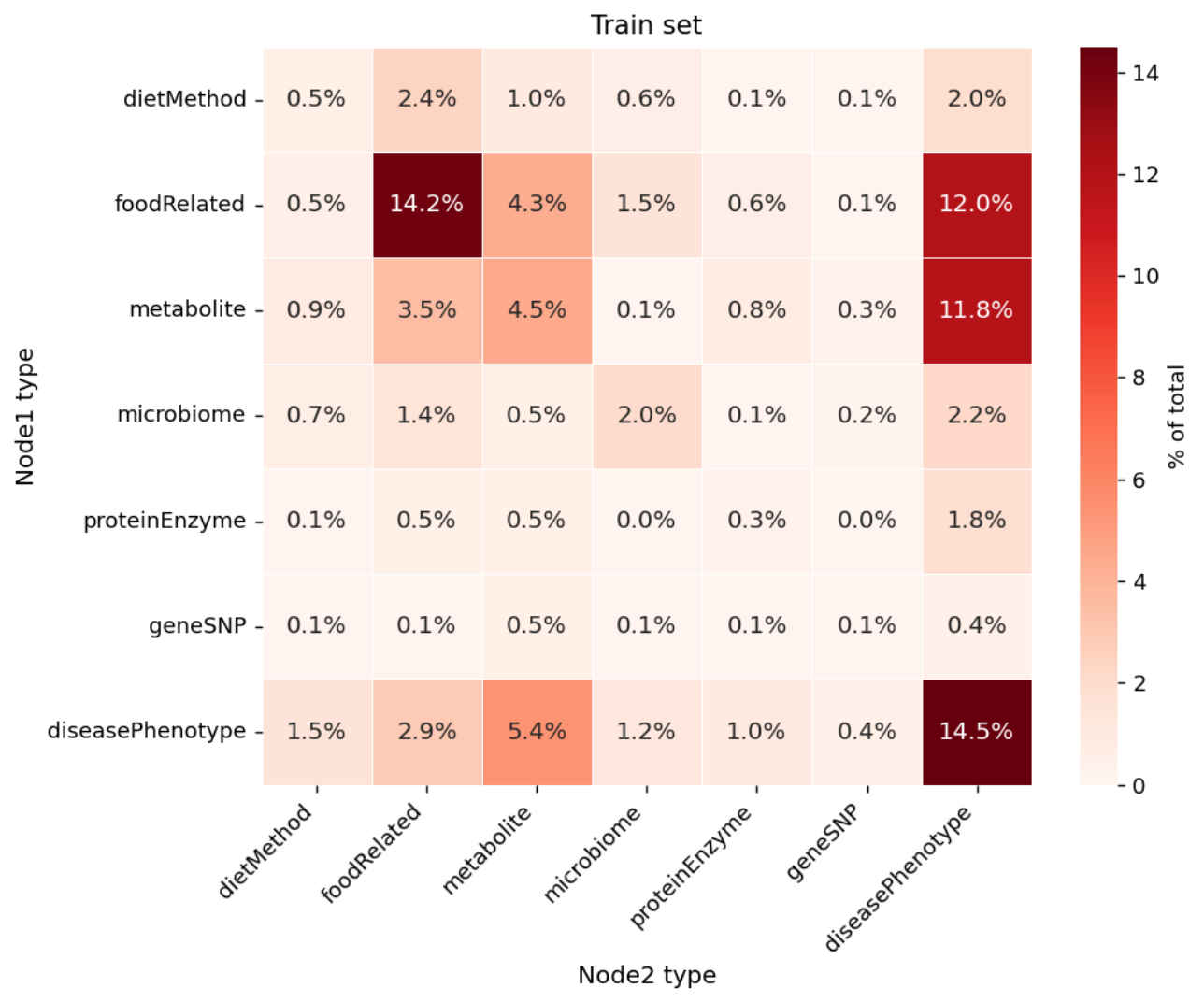

(a) Train split

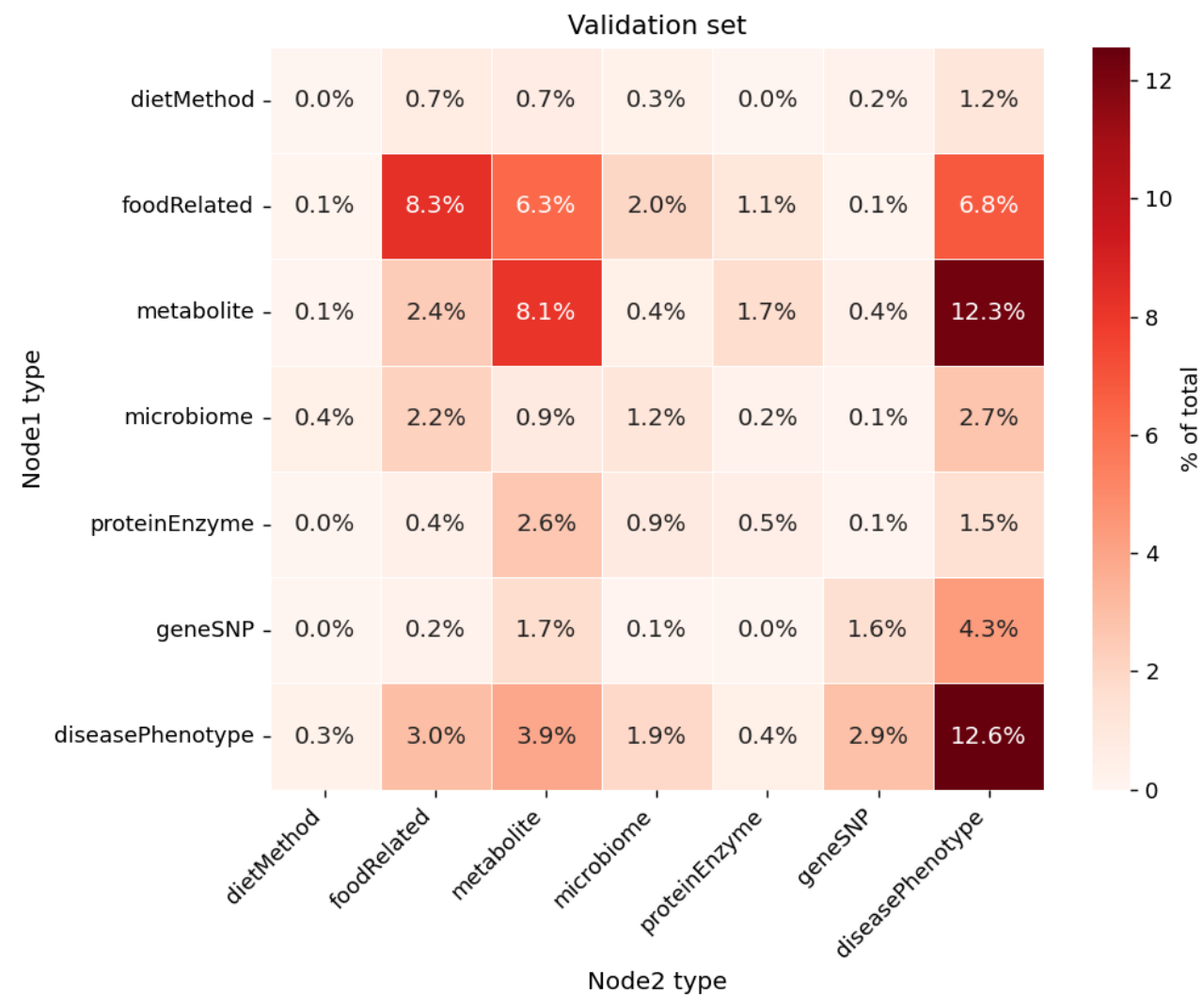

(b) Validation split

Figure S3 Graphical visualisation of CoCoS scores for a small set of relations across 4,445 documents from the CoDiet corpus (Lain et al., 2025). Node size is proportional to the number of documents where each node is part of in a relation. Node colour relates to the entity category. Edge width is proportional to the number of documents that reports the relation. Edge colour relates to the Effect Estimate (Eqn. 2).

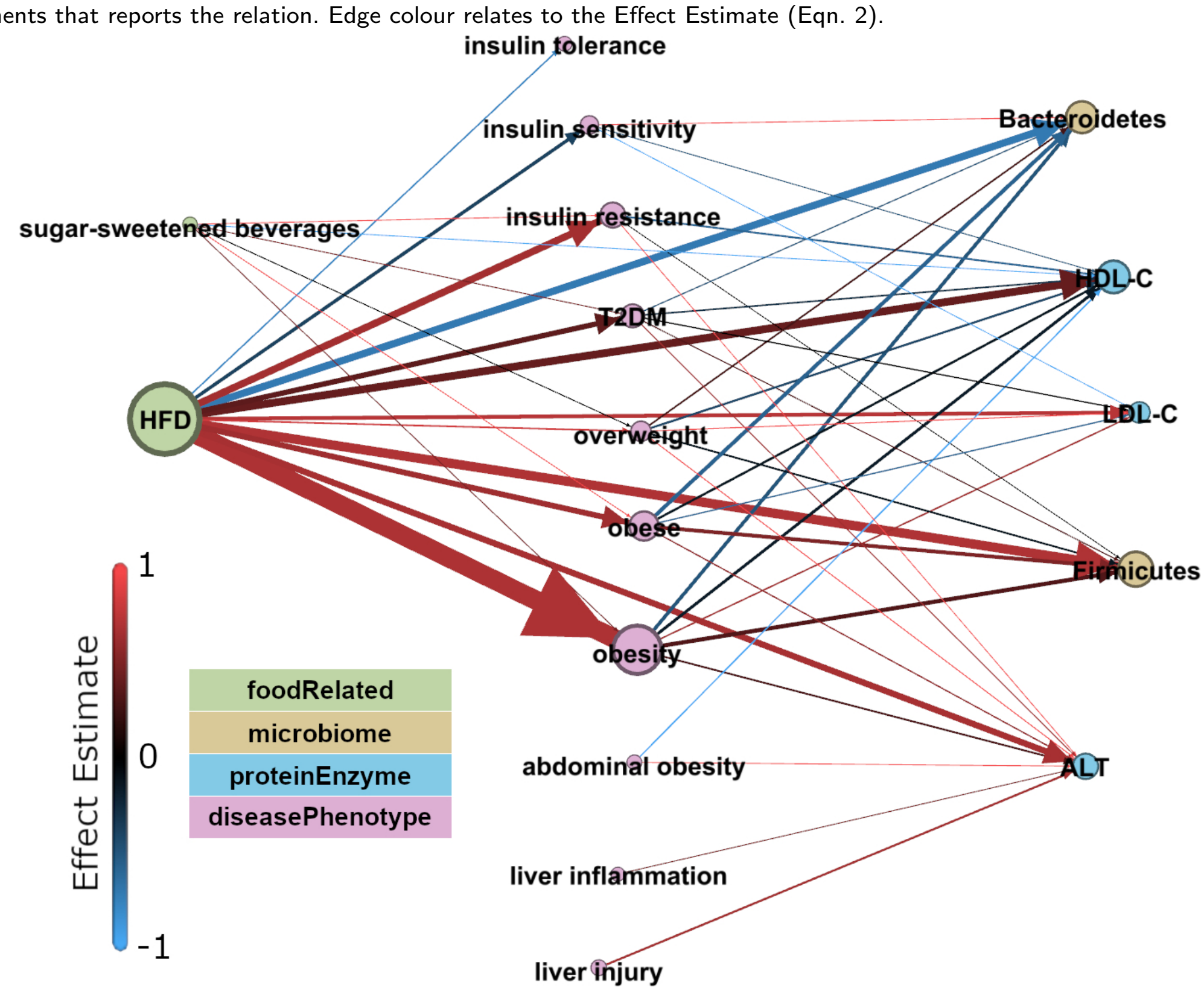

References

S. Agarwal, L. Ahmad, J. Ai, S. Altman, A. Applebaum, E. Arbus, R. K. Arora, Y. Bai, B. Baker, H. Bao, et al. gpt-oss-120b & gpt-oss-20b model card. *arXiv preprint arXiv:2508.10925*, 2025.

J. Devlin, M.-W. Chang, K. Lee, and K. Toutanova. Bert: Pre-training of deep bidirectional transformers for language understanding. In *Proceedings of the 2019 conference of the North American chapter of the association for computational linguistics: human language technologies, volume 1 (long and short papers)*, pages 4171–4186, 2019.

A. Dubey, A. Jauhri, A. Pandey, A. Kadian, A. Al-Dahle, A. Letman, A. Mathur, A. Schelten, A. Yang, A. Fan, et al. The llama 3 herd of models. *arXiv e-prints*, pages arXiv–2407, 2024.

Y. Gu, R. Tinn, H. Cheng, M. Lucas, N. Usuyama, X. Liu, T. Naumann, J. Gao, and H. Poon. Domain-specific language model pretraining for biomedical natural language processing, 2020.

D. Guo, D. Yang, H. Zhang, J. Song, R. Zhang, R. Xu, Q. Zhu, S. Ma, P. Wang, X. Bi, et al. Deepseek-r1: Incentivizing reasoning capability in llms via reinforcement learning. *arXiv preprint arXiv:2501.12948*, 2025.

H. Kim, H. Hwang, J. Lee, S. Park, D. Kim, T. Lee, C. Yoon, J. Sohn, D. Choi, and J. Kang. Small language models learn enhanced reasoning skills from medical textbooks. *arXiv preprint arXiv:2404.00376*, 2024.

W. Kwon, Z. Li, S. Zhuang, Y. Sheng, L. Zheng, C. H. Yu, J. E. Gonzalez, H. Zhang, and I. Stoica. Efficient memory management for large language model serving with pagedattention. In *Proceedings of the ACM SIGOPS 29th Symposium on Operating Systems Principles*, 2023.

A. D. Lain, S. Go, A. Mahmud, S. Rajendra, A. Cano San Jose, K. Loupasaki, G. Theodoridis, M. Bizkarguenaga Uribiarte, Y. Gu, O. Deda, R. D. A. Conde, N. Embade, A. de Diego Rodriguez, N. Burguera, D. Rossiou, R. Gil Redondo, D. Gallou, I. Tueros, R. Velmurugan, V. Gkanali, M. Caro Burgos, P. Pousinis, G. Alektoridis, S. Arranz, N. Nikolopoulos, X. Yan, R. Fernandez Carrion, T. Rowlands, D. Choi, M. Rei, C. Cave-Ayland, A. D'Alessandro, The CoDiet Consortium, T. Beck, and J. M. Posma. Codiet corpus. *bioRxiv*, 2025. doi: 10.1101/2025.09.04.673740. URL https://doi.org/10.1101/2025.09.04.673740.

J. Lee, W. Yoon, S. Kim, D. Kim, S. Kim, C. H. So, and J. Kang. Biobert: a pre-trained biomedical language representation model for biomedical text mining. *Bioinformatics*, 36(4):1234–1240, 2020.

A. Sellergren, S. Kazemzadeh, T. Jaroensri, A. Kiraly, M. Traverse, T. Kohlberger, S. Xu, F. Jamil, C. Hughes, C. Lau, et al. Medgemma technical report. *arXiv preprint arXiv:2507.05201*, 2025.

G. Team, T. Mesnard, C. Hardin, R. Dadashi, S. Bhupatiraju, S. Pathak, L. Sifre, M. Rivière, M. S. Kale, J. Love, et al. Gemma: Open models based on gemini research and technology. *arXiv preprint arXiv:2403.08295*, 2024.

T. Wolf, L. Debut, V. Sanh, J. Chaumond, C. Delangue, A. Moi, P. Cistac, T. Rault, R. Louf, M. Funtowicz, et al. Huggingface's transformers: State-of-the-art natural language processing. *arXiv preprint arXiv:1910.03771*, 2019.

A. Yang, A. Li, B. Yang, B. Zhang, B. Hui, B. Zheng, B. Yu, C. Gao, C. Huang, C. Lv, et al. Qwen3 technical report. *arXiv preprint arXiv:2505.09388*, 2025.

M. Yasunaga, J. Leskovec, and P. Liang. LinkBERT: Pretraining language models with document links. In *ICML 2022 Workshop on Knowledge Retrieval and Language Models*, 2022. URL https://openreview.net/forum?id=mAIHKEcg-sN.

Table S3 Multi-class classification results for the validation set. The validation set was used to select the best models and approaches to evaluate with the independent test set based on the F1-score. The top model from each category (BERT-based, general LLM, biomedical LLM) for each metric is indicated in bold font, with the second best models underlined. We omit DiMB-RE with the hierarchical experts approach due to difficulties in modifying the original codebase.

BERT model name	Type	Accuracy	F1 (Macro)	Precision (Macro)	Recall (Macro)
Bert-base	Normal	0.6307	0.5224	0.5466	0.5210
BioBERT	Normal	0.7025	0.6358	0.6851	**0.6169**
PubmedBERT	Normal	0.7083	0.6276	0.6667	0.6145
BioLinkBERT	Normal	0.7147	**0.6440**	**0.8006**	0.6149
DiMB-RE	Normal	**0.7238**	0.2940	0.4262	0.2360
Bert-base	Hierarchical - Individual Experts	0.5326	0.3980	0.4500	0.4084
BioBERT	Hierarchical - Individual Experts	0.6158	0.4686	0.4813	0.4640
PubmedBERT	Hierarchical - Individual Experts	0.6724	0.5017	0.5085	0.5010
BioLinkBERT	Hierarchical - Individual Experts	0.6330	0.4613	0.4954	0.4655
DiMB-RE	Hierarchical - Individual Experts	-	-	-	-
**General LLM name**	**Type**	**Accuracy**	**F1 (Macro)**	**Precision (Macro)**	**Recall (Macro)**
Llama-3.1-8B	Zero-shot	0.3978	0.4268	**0.7406**	0.3923
Qwen2.5-32B	Zero-shot	0.5885	0.5925	0.6125	0.6584
DeepSeek-R1-Qwen-32B	Zero-shot	0.6079	0.5742	0.6229	0.6390
DeepSeek-R1-Llama-70B	Zero-shot	0.5921	0.5956	0.6528	0.6317
Llama-3.3-70B	Zero-shot	0.5713	0.5442	0.5778	0.5961
gpt-oss-20B	Zero-shot	0.6280	0.6357	0.6688	0.6707
Llama-3.1-8B	Hierarchical prompt	0.5634	0.4624	0.5001	0.4683
Qwen2.5-32B	Hierarchical prompt	0.6057	0.5880	0.6978	0.5953
DeepSeek-R1-Qwen-32B	Hierarchical prompt	0.6050	0.5833	0.6922	0.5896
DeepSeek-R1-Llama-70B	Hierarchical prompt	0.6229	0.5805	0.6615	0.5993
Llama-3.3-70B	Hierarchical prompt	0.5756	0.5926	0.6466	0.6275
gpt-oss-20B	Hierarchical prompt	0.5957	0.6104	0.6450	0.6630
Llama-3.1-8B	Hierarchical prompt + reflection	0.5792	0.4831	0.5379	0.4818
Qwen2.5-32B	Hierarchical prompt + reflection	0.6143	0.5913	0.7160	0.5916
DeepSeek-R1-Qwen-32B	Hierarchical prompt + reflection	0.6065	0.5683	0.6214	0.6316
DeepSeek-R1-Llama-70B	Hierarchical prompt + reflection	0.6373	0.5870	0.6840	0.5913
Llama-3.3-70B	Hierarchical prompt + reflection	0.6222	0.6298	0.6945	0.6373
gpt-oss-20B	Hierarchical prompt + reflection	**0.6459**	**0.6481**	0.6794	**0.6837**
**Biomedical LLM name**	**Type**	**Accuracy**	**F1 (Macro)**	**Precision (Macro)**	**Recall (Macro)**
Meerkat-8B	Zero-shot	0.3871	0.3542	0.6130	0.3812
Meerkat-70B	Zero-shot	0.5670	0.6186	0.6908	0.6266
MedGemma-27B	Zero-shot	0.5986	0.6225	**0.7133**	0.6158
Meerkat-8B	Hierarchical prompt	0.4674	0.4039	0.4963	0.4167
Meerkat-70B	Hierarchical prompt	0.5728	0.5992	0.6202	0.6531
MedGemma-27B	Hierarchical prompt	0.6401	0.5526	0.5810	0.5672
Meerkat-8B	Hierarchical prompt + reflection	0.4552	0.3571	0.4981	0.3669
Meerkat-70B	Hierarchical prompt + reflection	0.6323	**0.6485**	0.6675	**0.6844**
MedGemma-27B	Hierarchical prompt + reflection	**0.6487**	0.6033	0.6997	0.5971

Table S4 Per-class distribution of RECoDe relation instances for the publicly released train and validation splits. The test split (49 documents) is reserved for the Codabench leaderboard and its per-class counts and percentages are not reported here to prevent test-set leakage. Overall corpus totals (train+validation+test combined) are reported in Table 1 of the main paper.

Class	Train	Validation
increaseAssociation	175 (11.7%)	191 (13.7%)
decreaseAssociation	225 (15.0%)	195 (14.0%)
association	309 (20.6%)	301 (21.6%)
causalEffect	233 (15.5%)	172 (12.3%)
substitution	12 (0.8%)	4 (0.3%)
consists	268 (17.9%)	253 (18.1%)
positiveCorrelation	35 (2.3%)	77 (5.5%)
negativeCorrelation	24 (1.6%)	28 (2.0%)
NoAssociation	95 (6.3%)	117 (8.4%)
Unrelated	124 (8.3%)	57 (4.1%)
**Total**	**1500**	**1395**

Table S5 Per-class F1 for the multi-class classification on the validation set, reported for six of the selected best-performing model setups (BioBERT, BioLinkBERT, Meerkat-70B, MedGemma-27B, Llama-3.3-70B, gpt-oss-20B) from Table 2 of the main paper. Top result for each class is given in bold font, and second best is underlined.

Class	BioBERT	BioLinkBERT	Meerkat-70B	MedGemma-27B	Llama-3.3-70B	gpt-oss-20B
increaseAssociation	<u>0.6993</u>	**0.7452**	0.6652	0.4765	0.5924	0.6369
decreaseAssociation	0.7848	**0.8081**	0.7578	0.7531	0.7045	<u>0.7957</u>
association	**0.6417**	<u>0.6407</u>	0.5618	0.5521	0.5576	0.5341
causalEffect	**0.6500**	<u>0.5993</u>	0.5751	0.3826	0.3894	0.5180
substitution	0.0000	0.0000	**0.8571**	**0.8571**	**0.8571**	0.7500
consists	0.7360	0.7505	0.6047	0.7353	<u>0.7982</u>	**0.8035**
positiveCorrelation	0.6569	**0.8000**	0.6625	0.6479	0.6944	<u>0.7482</u>
negativeCorrelation	**0.9057**	<u>0.7059</u>	0.5208	0.6173	0.4524	0.4595
NoAssociation	0.8219	0.8251	**0.8936**	0.8390	0.8517	<u>0.8826</u>
Unrelated	<u>0.4615</u>	**0.5647**	0.3864	0.3636	0.4000	0.3521
**macro F1**	0.6358	0.6440	**0.6485**	0.6225	0.6298	<u>0.6481</u>

Prompts

This section provides the full prompts used at each stage of the RECoDe relation extraction decision flow (Fig. 2). Each prompt corresponds to a specific classifier (C1–C7) in the staged framework and implements the branching logic defined in the decision process.

Figure S4. Global system instruction used to initialise the RECoDe relation extraction decision flow (Fig. 2). This prompt establishes the clinical reviewer role and defines the scientific reasoning context that guides all subsequent staged classification prompts (C1–C7) within the hierarchical RECoDe decision framework.

```
You are a Clinical Scientific Reviewer specializing in biomedical relation extraction.
Your task is to analyze given texts and extract meaningful relationships
between biomedical entities. Provide accurate, concise, and well-structured
explanations based on scientific principles.
```

Figure S5. Prompt used in the C1First stage of the RECoDe decision flow (Fig. 2) to separate negative cases (NoAssociation and Unrelated) from positive relations, where Yes predictions are mapped to the C2Asso branch.

```
Determine whether there is a 'association' relationship between '[ENTITY1]' and '[ENTITY2]' in the following sentence: '[SENTENCE]'.

Answer only with one of the following options:
(A) Yes - '[ENTITY1]' and '[ENTITY2]' have a **direct** association (causation, correlation, substitution, inclusion, or
↪  biological/chemical interaction).
(B) NoAssociation - The sentence explicitly states that '[ENTITY1]' and '[ENTITY2]' have **no effect** on each other, or '[ENTITY1]' is
↪  **excluded from** '[ENTITY2]'.
(C) Unrelated - '[ENTITY1]' and '[ENTITY2]' are **mentioned together** but without any meaningful connection.

**Guidelines for classification:**
- Select **(B) NoAssociation** if the sentence explicitly states that there is **no effect**, **no impact**, or **no causal
↪  relationship** between '[ENTITY1]' and '[ENTITY2]'.
- Select **(B) NoAssociation** if '[ENTITY1]' is explicitly **excluded, bypassed, or omitted** from '[ENTITY2]' (e.g., 'privation from',
↪  'not included in', 'bypassing', 'X-free', 'avoids', etc.).
- Select **(C) Unrelated** if '[ENTITY1]' and '[ENTITY2]' are simply mentioned together but **without** any logical or meaningful
↪  relationship.

**Examples of when to answer (B) NoAssociation:**
- The sentence explicitly states that '[ENTITY1]' **does not affect** '[ENTITY2]' in any way.
- The sentence concludes that **there is no causal or meaningful relationship** between '[ENTITY1]' and '[ENTITY2]'.
- A study or analysis is mentioned, but it states **no significant association** between '[ENTITY1]' and '[ENTITY2]'.
- The sentence implies a possible connection but **ultimately dismisses it** as insignificant.
- '[ENTITY1]' is **excluded, omitted, or bypassed** in relation to '[ENTITY2]' (e.g., 'X-free diet excludes Y', 'X avoids Y', 'X is
↪  omitted from Y').

**Examples of when to answer (C) Unrelated:**
- '[ENTITY1]' and '[ENTITY2]' are mentioned **in the same list**, but there is **no stated relationship**.
- The sentence describes characteristics of '[ENTITY1]' and '[ENTITY2]' separately without linking them.
- '[ENTITY1]' and '[ENTITY2]' belong to the same category (e.g., both are chemicals, foods, or medical terms) but are not directly
↪  connected.
- The sentence compares '[ENTITY1]' and '[ENTITY2]' (e.g., one is healthier than the other) but **does not describe any direct
↪  interaction**.

**Answer (A) Yes ONLY IF:**
- The sentence explicitly states that '[ENTITY1]' and '[ENTITY2]' **have a direct association**, such as **causation, correlation,
↪  substitution, or biological/chemical interaction**.
- '[ENTITY1]' and '[ENTITY2]' are part of the **same biological or functional process** with an explicitly stated relationship.
- One entity is a **component, ingredient, or essential part** of the other.
- '[ENTITY1]' and '[ENTITY2]' directly affect each other in a **positive or negative direction**.

**Examples of when to answer (A) Yes:**
- '[ENTITY1]' and '[ENTITY2]' have a **direct or indirect effect** on each other.
- The sentence explicitly states that '[ENTITY1]' and '[ENTITY2]' are related through **substitution, correlation, or causation**.
- '[ENTITY1]' and '[ENTITY2]' are part of the same **biological/metabolic pathway** with a clear, explicit relationship.
- One entity is **necessary for the function of the other**.

**Provide your answer in the following format:**
Reason: Provide an explanation focusing on key verbs, negations, and the relationship between '[ENTITY1]' and '[ENTITY2]'.
```

```
Answer: (A) Yes OR (B) NoAssociation OR (C) Unrelated
```

Figure S6. Prompt used in the C2Asso stage of the RECoDe decision flow (Fig. 2) to categorise relations from C1First into Correlation, Association, or Contains branches, which are subsequently routed to the C3Corr, C4MiddleAsso, and C6ContainSubsti stages, respectively.

```
Determine the broad category of relationship between '[ENTITY1]' and '[ENTITY2]' in the following sentence: '[SENTENCE]'.

Answer only with one of the following options:
(A) Correlation - '[ENTITY1]' and '[ENTITY2]' show a statistical or directional relationship (e.g., positive or negative correlation).
(B) Association - '[ENTITY1]' and '[ENTITY2]' have a direct connection (e.g., causal effect, increase, decrease, or influence).
(C) Contains - '[ENTITY1]' contains, includes, or substitutes '[ENTITY2]'.

Guidelines for classification:
- (A) Correlation: Use this if '[ENTITY1]' and '[ENTITY2]' are correlated (either positively or negatively) but without direct influence.
- (B) Association: Use this if '[ENTITY1]' has a direct effect on '[ENTITY2]', increases, decreases, or influences it in any way.
- (C) Contains: Use this if '[ENTITY1]' consists of, includes, or substitutes '[ENTITY2]'. If '[ENTITY1]' replaces or is replaced by
↪  '[ENTITY2]', use Contains.

**Key distinctions:**
- Choose **(B) Association** if '[ENTITY1]' leads to or implies a higher/lower level or likelihood of '[ENTITY2]', or vice versa
↪  ('[ENTITY2]' to '[ENTITY1]'), even if the sentence uses passive voice or indirect expressions.
- Choose **(A) Correlation** only when the sentence mentions a co-occurrence or relationship between '[ENTITY1]' and '[ENTITY2]' without
↪  implying any directional effect.
- Choose **(C) Contains** if one entity includes, consists of, or substitutes the other.

- If the sentence explicitly states that '[ENTITY1]' and '[ENTITY2]' are correlated but does not specify causation, choose **(A)
↪  Correlation**.
- If '[ENTITY1]' is said to influence, increase, or decrease '[ENTITY2]', choose **(B) Association**.
- If '[ENTITY1]' is replacing, being replaced by, or is a substitute for '[ENTITY2]', choose **(C) Contains**.

Examples:
- (A) Correlation: 'Higher X levels are associated with higher/lower Y levels.' OR 'When X levels are low, some gene increases Y levels'
- (B) Association: 'X causes Y' OR 'X increases/decreases Y' OR 'X supplementation leads to a reduction in Y levels.' OR 'X had a higher
↪  prevalence of B'.
- (C) Contains: 'X is made up of Y' OR 'X is a substitute for Y' OR 'X is replaced with Y'.

Provide your answer in the following format:
Reason: Explain the relationship based on key verbs and negations if any.
Answer: (A) Correlation OR (B) Association OR (C) Contains
```

Figure S7. Prompt used in the C3Corr stage of the RECoDe decision flow (Fig. 2) to distinguish between PositiveCorrelation and NegativeCorrelation, with PositiveCorrelation predictions routed to the C7PositiveCorrReflection stage. The decision flow uses lowercase labels for consistency with the annotation schema, whereas the prompt presents capitalised forms as model output options.

```
Determine the **specific type of correlation** between '[ENTITY1]' and '[ENTITY2]' in the following sentence: '[SENTENCE]'.

Answer only with one of the following options:
(A) PositiveCorrelation - '[ENTITY1]' and '[ENTITY2]' increase or decrease together.
(B) NegativeCorrelation - When '[ENTITY1]' increases, '[ENTITY2]' decreases (or vice versa).

**Key distinctions:**
- If '[ENTITY1]' and '[ENTITY2]' increase or decrease together, choose **(A) PositiveCorrelation**.
- If '[ENTITY1]' increases while '[ENTITY2]' decreases (or vice versa), choose **(B) NegativeCorrelation**.

**Examples:**
- (A) PositiveCorrelation: 'Higher X intake is associated with higher Y levels.'
- (B) NegativeCorrelation: 'As X levels increase, Y levels decrease.'

Provide your answer in the following format:
Reason: Explain the relationship based on key verbs and directionality.
Answer: (A) PositiveCorrelation OR (B) NegativeCorrelation
```

Figure S8. Prompt used in the C4MiddleAsso stage of the RECoDe decision flow (Fig. 2) to classify association relations into IncreaseAssociation, DecreaseAssociation, or GeneralAssociation, where GeneralAssociation predictions are routed to the C5AssoCausal stage. The decision flow uses lowercase labels for consistency with the annotation schema, whereas the prompt presents capitalised forms as model output options.

```
Determine the specific type of association between '[ENTITY1]' and '[ENTITY2]' in the following sentence: '[SENTENCE]'.

Answer only with one of the following options:
(A) IncreaseAssociation - '[ENTITY1]' increases or enhances '[ENTITY2]'.
(B) DecreaseAssociation - '[ENTITY1]' decreases or inhibits '[ENTITY2]'.
(C) GeneralAssociation - '[ENTITY1]' and '[ENTITY2]' are related, but the nature of the effect is unclear (e.g., general association,
↪  causal relationship without specifying increase or decrease).

**Guidelines for classification:**
- (A) IncreaseAssociation: Choose this if '[ENTITY1]' increases, enhances, or boosts '[ENTITY2]'.
- Use (A) if '[ENTITY1]' leads to or is linked with **higher** levels, prevalence, or activity of '[ENTITY2]'.
- (B) DecreaseAssociation: Choose this if '[ENTITY1]' decreases, inhibits, or suppresses '[ENTITY2]'.
- These directional cues may be **inside or outside the entity span**, and may appear as verbs (e.g., 'increases', 'reduces') or
↪  adjectives/nouns (e.g., 'higher', 'lower', 'reduction in').
- Use (B) if '[ENTITY1]' leads to or is linked with **lower** levels, prevalence, or activity of '[ENTITY2]'.
- (C) GeneralAssociation: Choose this if '[ENTITY1]' and '[ENTITY2]' are related but the sentence does not explicitly state an increase
↪  or decrease effect.
Use (C) GeneralAssociation if the sentence describes a **causal relationship** (e.g., '[ENTITY1]' causes '[ENTITY2]') without specifying
↪  whether it increases or decreases.
- Use (C) only if there's no directional cue or causal wording.

**Examples:**
- (A) IncreaseAssociation: 'X supplementation leads to increased Y levels.'
- (A) IncreaseAssociation: 'Obesity is associated with higher hypertension rates.'
- (B) DecreaseAssociation: 'Folate supplementation leads to a reduction in homocysteine levels.'
- (B) DecreaseAssociation: 'X intake reduces Y levels significantly.'
- (C) GeneralAssociation: 'X and Y are linked in metabolic pathways.'

- (C) GeneralAssociation: 'X causes Y in a biological process.' (without indication of increase or decrease)
- (C) GeneralAssociation: 'X leads to Y without stating whether it increases or decreases.'

**Provide your answer in the following format:**
Reason: Explain the relationship, focusing on key verbs and negations if any.
Answer: (A) IncreaseAssociation OR (B) DecreaseAssociation OR (C) GeneralAssociation
```

Figure S9. Prompt used in the C5AssoCausal stage of the RECoDe decision flow (Fig. 2) to distinguish between CausalEffect and Association for relations routed from the C4MiddleAsso stage. The decision flow uses lowercase labels for consistency with the annotation schema, whereas the prompt presents capitalised forms as model output options.

```
Determine whether the relationship between '[ENTITY1]' and '[ENTITY2]' in the following sentence is a **direct causal effect** or a
↪  **general association**: '[SENTENCE]'

Answer only with one of the following options:
(A) CausalEffect - '[ENTITY1]' **directly causes, leads to, results in, or significantly alters** '[ENTITY2]'.
(B) Association - '[ENTITY1]' and '[ENTITY2]' are related, but causation is **not explicitly stated**.

**Key distinctions:**
- Choose **(A) CausalEffect** if the sentence explicitly states that '[ENTITY1]' **directly causes** '[ENTITY2]'. Common indicators:
↪  'causes', 'results in', 'leads to', 'is responsible for', 'induces', 'triggers', 'generates', 'significantly alters', 'plays a key
↪  role in'.
- Choose **(B) Association** if '[ENTITY1]' and '[ENTITY2]' are linked but the direction of causality is not certain. Common indicators:
↪  'associated with', 'linked to', 'correlated with', 'influences', 'impacts', 'modifies'.

**Additional rule:**
- If the sentence **strongly implies a cause-effect relationship** without explicitly stating it, but the mechanism is known from
↪  scientific literature, choose **(A) CausalEffect**.
- If the sentence only states a statistical or observational link, choose **(B) Association**.
- If '[ENTITY1]' is described as a **risk factor** for '[ENTITY2]' it should be labeled as **(B) Association**.

**Examples:**
- (A) CausalEffect: 'Obesity **leads to** increased blood pressure.'
- (A) CausalEffect: 'High salt intake **induces** hypertension by increasing water retention.'
- (A) CausalEffect: 'A ketogenic diet **significantly alters** lipid metabolism.'
- (B) Association: 'Obesity **is a major risk factor** for hypertension.'
```

```
- (B) Association: 'Obesity **is associated with** increased blood pressure.'
- (B) Association: 'People who consume more salt **tend to** have higher blood pressure.'

Provide your answer in the following format:
Reason: Explain the relationship based on key verbs and causality.
Answer: (A) CausalEffect OR (B) Association
```

Figure S10. Prompt used in the C6ContainSubsti stage of the RECoDe decision flow (Fig. 2) to distinguish between Consists and Substitution relations for cases routed from the C2Asso Contains branch. The decision flow uses lowercase labels for consistency with the annotation schema, whereas the prompt presents capitalised forms as model output options.

```
Determine the **specific type of 'contains' relationship** between '[ENTITY1]' and '[ENTITY2]' in the following sentence: '[SENTENCE]'.

Answer only with one of the following options:
(A) Consists - '[ENTITY1]' **is made up of, includes, or contains** '[ENTITY2]'.
(B) Substitution - '[ENTITY1]' **replaces or is replaced by** '[ENTITY2]'.

**Key distinctions:**
- Choose **(A) Consists** if '[ENTITY1]' contains '[ENTITY2]' as an ingredient, component, or part.
- Choose **(B) Substitution** if '[ENTITY1]' and '[ENTITY2]' are alternatives to each other, meaning one replaces or is replaced by the
↪ other.

**Additional rule:**
- If the sentence **describes '[ENTITY1]' as containing '[ENTITY2]'**, such as an ingredient in food, a molecule in a compound, or a
↪ component in a system, choose **(A) Consists**.
- If the sentence **describes '[ENTITY1]' being swapped or exchanged for '[ENTITY2]'**, such as in dietary replacements or drug
↪ alternatives, choose **(B) Substitution**.

**Examples:**
- (A) Consists: 'Milk **contains** calcium.'
- (A) Consists: 'Fish oil **is rich in** omega-3 fatty acids.'
- (A) Consists: 'Wheat flour **is a source of** gluten.'
- (B) Substitution: 'Butter **can be replaced with** margarine in this recipe.'
- (B) Substitution: 'Plant-based protein **is an alternative to** animal protein.'
- (B) Substitution: 'Artificial sweeteners **substitute for** sugar in many processed foods.'

Provide your answer in the following format:
Reason: Explain the relationship based on key verbs and the nature of the relationship.
Answer: (A) Consists OR (B) Substitution
```

Figure S11. Reflection prompt used in the C7PositiveCorr stage of the RECoDe decision flow (Fig. 2) to re-evaluate PositiveCorrelation predictions, confirming PositiveCorrelation or revising them to IncreaseAssociation or Association when appropriate. The decision flow uses lowercase labels for consistency with the annotation schema, whereas the prompt presents capitalised forms as model output options.

```
The previous analysis classified the relationship between '[ENTITY1]' and '[ENTITY2]' in the following sentence as
↪ **PositiveCorrelation**.

Sentence: [SENTENCE]

Please re-evaluate the classification.

Choose one of the following options:
(A) Confirm to PositiveCorrelation - Both entities tend to **increase or decrease together**, either across time or under specific
↪ conditions or groups. This includes phrases like 'correlated with' or 'positively correlated with'. In cases such as 'positively
↪ associated with' or 'linked to higher levels of', choose this only **if both '[ENTITY1]' and '[ENTITY2]' are clearly described as
↪ increasing (e.g., 'high', 'higher', 'elevated') or decreasing (e.g., 'low', 'lower') in the same direction**. Directionality can also
↪ be **implicit**, such as numeric trends (e.g., '10mg to 20mg'). If such co-directional change is not clearly described for both
↪ entities, consider other options instead.

(B) Revise to IncreaseAssociation - One entity is observed or reported to be at a **higher level when the other is present**, even if
↪ causality is not explicitly stated. This includes expressions such as '[ENTITY1] was significantly higher in ENTITY2]', or numeric
↪ comparisons that suggest an increase (e.g., from 10mg to 20mg). Also apply this when an increase is **implicitly indicated** through
↪ phrases like 'elevated levels', 'upregulated in group X', or similar comparative language.

(C) Revise to Association - The two are associated, but the **directional or co-variation pattern** is not clear enough.
```

Guidelines:
- Choose (A) if both entities clearly increase/decrease together (e.g., correlated or co-varying patterns).
- Choose (B) if one entity increases (or is higher) **in the presence or context of** the other.
- Choose (C) if the relationship is vague or lacks clear directionality.

Examples:
- (A) Confirm to PositiveCorrelation - 'Higher levels of X are associated with higher levels of Y.'
- (A) Confirm to PositiveCorrelation - '<entity1>Firmicutes/Bacteroidetes ratio</entity1> significantly correlated positively with <entity2>total cholesterol</entity2>.'
- (B) Revise to IncreaseAssociation - 'X increases Y levels significantly.'
-(B) Revise to IncreaseAssociation - 'The mean value of the <entity1>C-reactive protein</entity1> had the highly significant highest value among the <entity2>obese</entity2> women without MetS.'
- (C) Revise to Association - 'The <entity1>gut microbiota</entity1> is associated with <entity2>obesity</entity2>.'

Provide your answer in the following format:
Reason: Briefly explain the reasoning using key phrases or structures in the sentence.
Answer: (A) Confirm to PositiveCorrelation OR (B) Revise to IncreaseAssociation OR (C) Revise to Association
